# Genomic Evidence for Island Population Conversion Resolves Conflicting Theories of Polar Bear Evolution

**DOI:** 10.1371/journal.pgen.1003345

**Published:** 2013-03-14

**Authors:** James A. Cahill, Richard E. Green, Tara L. Fulton, Mathias Stiller, Flora Jay, Nikita Ovsyanikov, Rauf Salamzade, John St. John, Ian Stirling, Montgomery Slatkin, Beth Shapiro

**Affiliations:** 1 Department of Ecology and Evolutionary Biology, University of California Santa Cruz, Santa Cruz, California, United States of America; 2 Department of Biomolecular Engineering, University of California Santa Cruz, Santa Cruz, California, United States of America; 3 Department of Integrative Biology, University of California Berkeley, Berkeley, California, United States of America; 4 Wrangel Island State Nature Reserve, Pevek, Russia; 5 Wildlife Research Division, Department of Environment, Edmonton, Canada; 6 Department of Biological Sciences, University of Alberta, Edmonton, Canada; University of Arizona, United States of America

## Abstract

Despite extensive genetic analysis, the evolutionary relationship between polar bears (*Ursus maritimus*) and brown bears (*U. arctos*) remains unclear. The two most recent comprehensive reports indicate a recent divergence with little subsequent admixture or a much more ancient divergence followed by extensive admixture. At the center of this controversy are the Alaskan ABC Islands brown bears that show evidence of shared ancestry with polar bears. We present an analysis of genome-wide sequence data for seven polar bears, one ABC Islands brown bear, one mainland Alaskan brown bear, and a black bear (*U. americanus*), plus recently published datasets from other bears. Surprisingly, we find clear evidence for gene flow from polar bears into ABC Islands brown bears but no evidence of gene flow from brown bears into polar bears. Importantly, while polar bears contributed <1% of the autosomal genome of the ABC Islands brown bear, they contributed 6.5% of the X chromosome. The magnitude of sex-biased polar bear ancestry and the clear direction of gene flow suggest a model wherein the enigmatic ABC Island brown bears are the descendants of a polar bear population that was gradually converted into brown bears via male-dominated brown bear admixture. We present a model that reconciles heretofore conflicting genetic observations. We posit that the enigmatic ABC Islands brown bears derive from a population of polar bears likely stranded by the receding ice at the end of the last glacial period. Since then, male brown bear migration onto the island has gradually converted these bears into an admixed population whose phenotype and genotype are principally brown bear, except at mtDNA and X-linked loci. This process of genome erosion and conversion may be a common outcome when climate change or other forces cause a population to become isolated and then overrun by species with which it can hybridize.

## Author Summary

The evolutionary genetic relationship between polar bears (*Ursus maritimus*) and brown bears (*U. arctos*) is a subject of continuing controversy. To address this we generated genome-wide sequence data for seven polar bears, two brown bears (including one from the enigmatic ABC Islands population), and a black bear (*U. americanus*). These data reveal remarkable genetic homogeneity within polar bears and clear evidence of past hybridization with brown bears. Hybridization, however, appears to be limited to habitat islands, where isolated populations of polar bears are gradually converted into brown bears via male-mediated dispersal and sex-biased gene flow. Our simplified and comprehensive model for the origin and evolution of polar bears resolves conflicting interpretations of mitochondrial and nuclear genetic data, and highlights the potential effect of natural climate change on long-term evolutionary processes.

## Introduction

**Figure 1:**
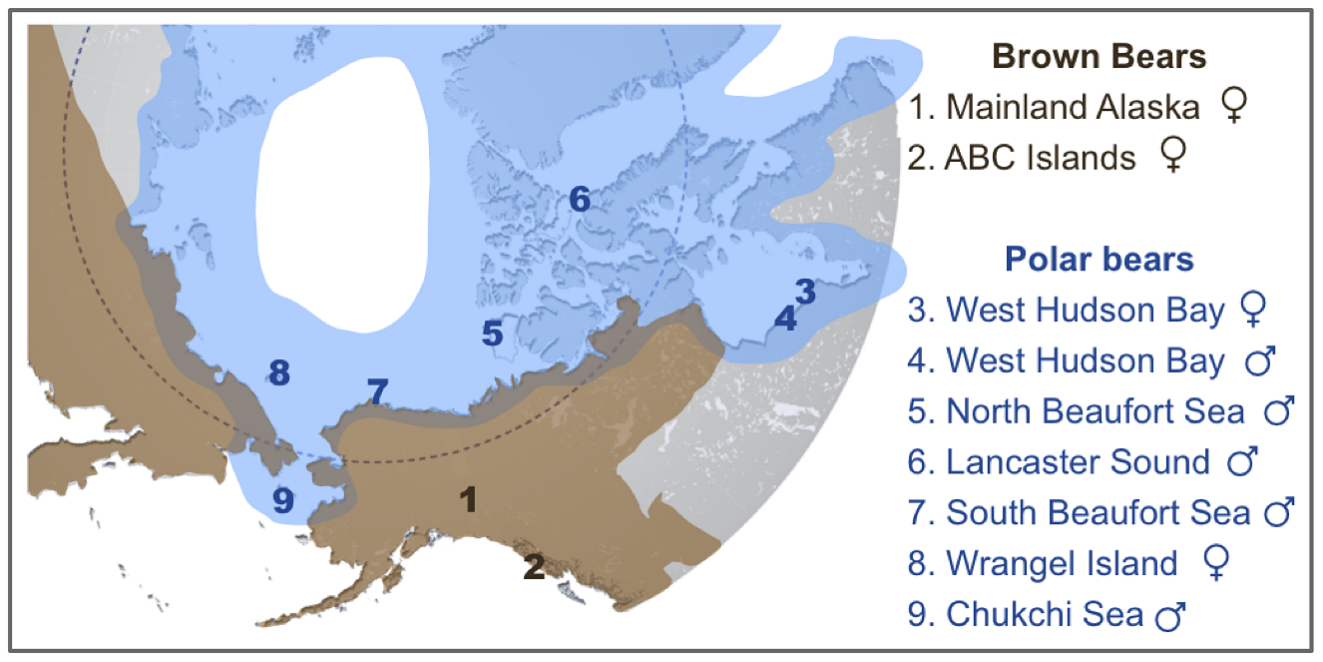
Map showing the approximate current geographic ranges of brown bears (brown) and polar bears (blue). Numbers indicate the geographic location of origin of two brown bears and seven polar bears analyzed here. An American black bear from central Pennsylvania was also sequenced as part of this study. Shotgun data amounting to 4–6× coverage for polar bears and 11–12× coverage for brown and black bears (Table S2) was aligned to the current distribution of the polar bear genome [13].

Despite polar bears' clear morphological and behavioral adaptations to their arctic environment [1], [2], their genetic relationship to brown bears remains unclear [3], [4], [5], [6]. Analysis of maternally inherited mitochondrial DNA (mtDNA) shows that polar bears fall within the range of variation of brown bears. Extant brown bears from Alaska's ABC (Admiralty, Baranof and Chichagof) Islands, some extinct brown bears from Ireland and mainland Alaska, and two ∼115,000-year-old polar bears share the mtDNA haplotype of all extant polar bears [3], [7], [8], [9], [10], [11]. The time to most recent common ancestor (TMRCA) of this mtDNA haplotype has been estimated at ∼160 thousand years ago (kya) (Figure S7) [3], [6], [9], [10]. Recent analysis of data from a panel of brown and polar bears at 14 nuclear loci showed that polar bears are generally distinct from brown bears, with genomic TMRCA averaging ∼600 kya [4]. Under a simple population split model without subsequent admixture, the population divergence should be more recent than average genomic divergence and thus polar bears became a distinct species more recently than 600 kya. A separate recent genome sequencing survey concluded that brown bear and polar bear lineages are much older. Miller and colleagues concluded that the lineage that would become polar bears diverged from that which would become brown bears more than 4 million years ago, followed by admixture that continues to the present [6]. Consistent with this, the past and present geographic ranges of both species overlap at their margins (Figure 1), and fertile hybrids are known in both captive and wild populations [2], [12].

The current consensus is that mtDNA and perhaps other polar bear loci are the result of past introgressions from brown bears into polar bears [3], [4], [6]. One scenario that has been proposed to reconcile the complicated discordance between the mtDNA trees and the species trees requires at least two instances of hybridization [3]. The first, which must have occurred before ∼115kya, passed the mtDNA haplotype from polar bears into brown bears, including the ancient Irish brown bears and ancestors of the ABC Island brown bears. The second passed this mtDNA haplotype back into polar bears, after which it came to fixation in all extant polar bears. This convoluted scenario is necessary if, in fact, polar bears derive their mtDNA haplotype and other loci from brown bears. Unfortunately, this prevailing consensus has gone unquestioned. Here, we present an analysis of published and newly generated genome-wide data for brown bears and polar bears. We find extensive evidence of previous admixture, from polar bears into brown bears, especially of X-linked genes.

## Results

To more fully delineate the genetic relationship between polar bears and brown bears, we sequenced random genomic shotgun libraries from seven polar bears, two brown bears and one black bear to learn the ancestral state for alleles (Figure 1, Text S1). We mapped these reads to the assembled genome scaffolds of polar bear (Text S1) [13]. Because the sequence coverage of each bear was uneven and too low to reliably call heterozygous sites, we down-sampled the sequence data from each bear to 1×. That is, we randomly picked a high-quality base from amongst all reads that mapped reliably at each position in the bear genome. In this way, we generated a composite haplotype for each bear and used these data for further analysis.

**Figure 2:**
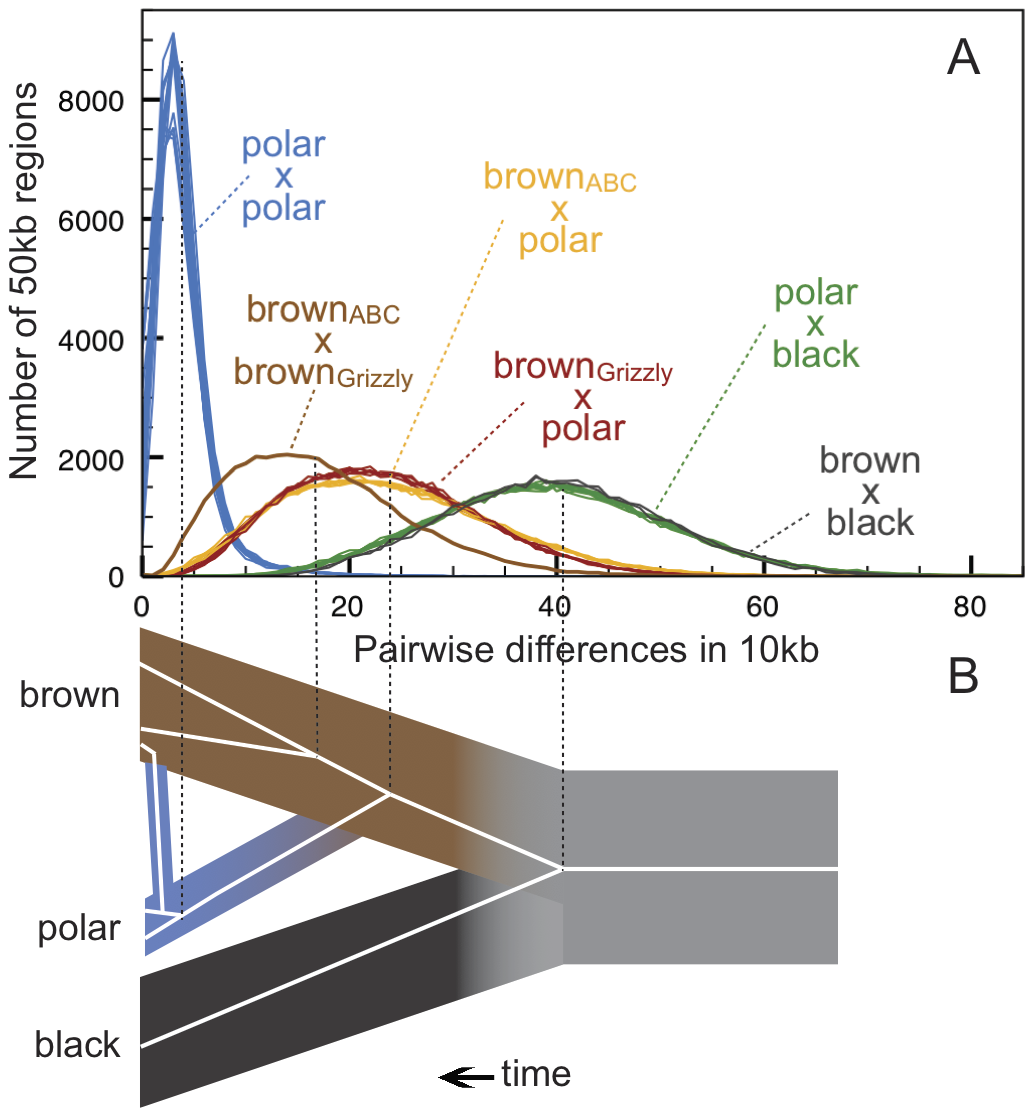
Genetic diversity within and between bear species. (A) Pairwise differences between individuals estimated as the average number of differences per 10 thousand bases (kb) in 42,000 non-overlapping 50 kb regions. After strict quality filtering, within-sample heterozygosity was resolved by selecting a single, high-quality base at random. The Lancaster Sound polar bear showed an excess of postmortem damage, as expected for historic specimens [32], and is shown in Figure S1. Polar bears are remarkably homogenous compared to brown bears, and both polar bears and brown bears are approximately equally diverged from the American black bear. Consistent with the results of the *D-*statistic test, pairwise distance between the ABC Islands brown bear and all polar bears (yellow lines) is less than that between the mainland brown bear and all polar bears (red lines). (B) Schematic diagram of a representative gene tree within brown bear, polar bear, and black bear populations, with the present day at the left of the diagram. For this locus, admixture occurring more recently than the population divergence of polar bears leads to the introgression of a polar bear haplotype into brown bears. Estimate of average genomic distance for brown, black, and polar bears and for population divergence between brown bears and polar bears given different calibration points are provided in Table 1.

To gauge the level of diversity within and divergence between bear species, we made pairwise comparison between each bear, in 50 kb windows, across the bear genome (Figure 2). In agreement with previous reports [4], [14], we find that polar bears are remarkably homogeneous: polar bear alleles differ at ∼4 sites in 10,000. In contrast, brown bears have roughly four times as much genetic diversity, differing at ∼17 in 10,000 sites. We note that the level of diversity among brown bears is nearly as high as the divergence between brown and polar bears. As expected, polar bears and brown bears show similar pairwise genomic divergence from the black bear. Likewise, the polar bears, brown bears, and black bears all show similar genomic divergence from the giant panda (*Ailuropoda melanoleuca*) [15].

**Figure 3:**
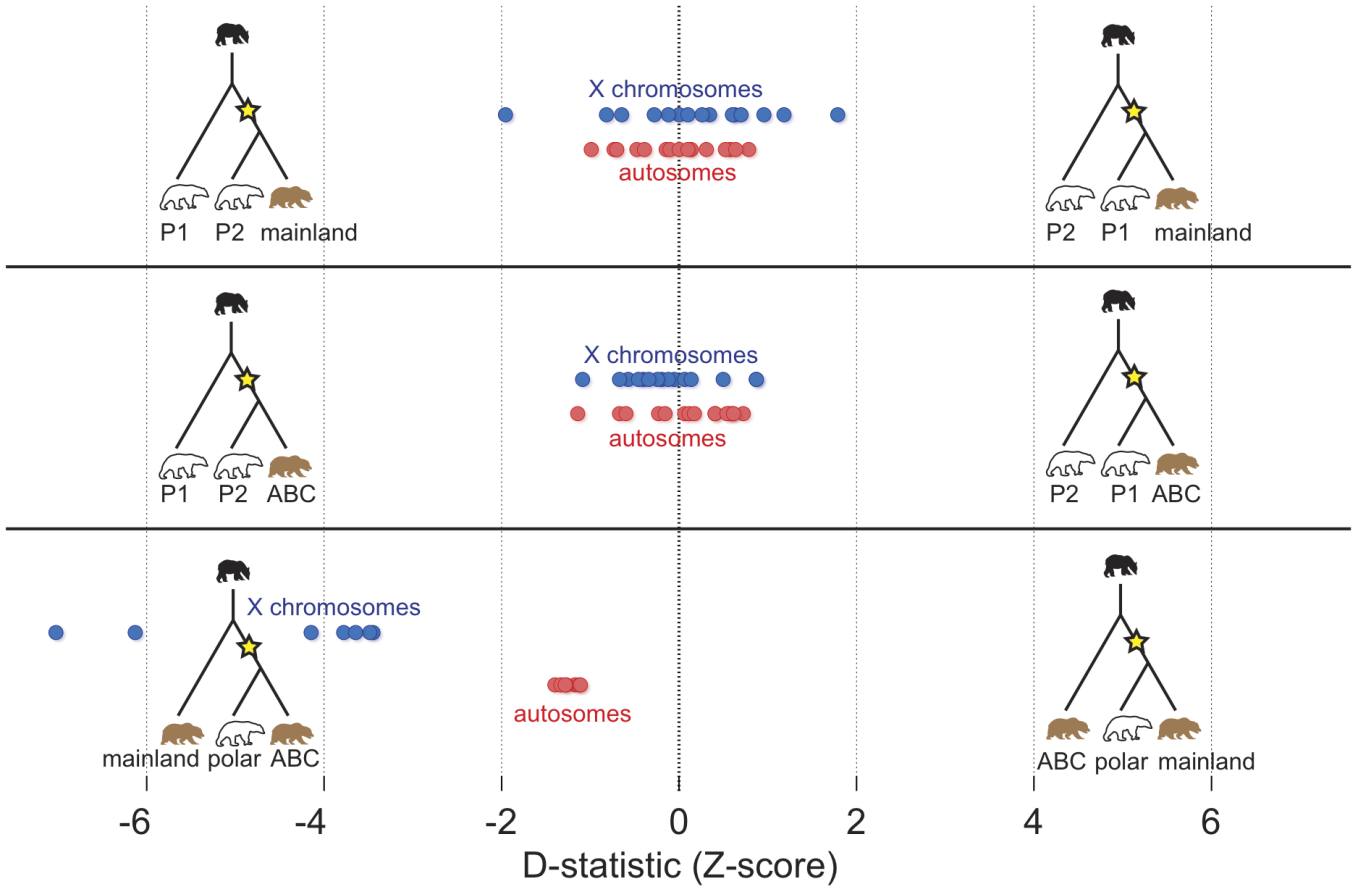
Summary of D-statistic comparisons between polar bears and brown bears. In each comparison, the black bear was used to define the ancestral allele. The Z-score of the D-statistic for each comparison is shown for autosomes (red) and X-chromosome (blue). Each dot represents the data from comparison of one pair of bears. In the top panel, all pairs of polar bears are compared for excess derived allele matching against the mainland brown bear. In the middle panel, all pairs of polar bears are compared against the ABC Island brown bear. The bottom panel shows the comparison of the two brown bears for excess allele matching to polar bears with each dot representing a different polar bear.

We quantified admixture between brown and polar bears using the *D*-statistic [16]. In brief, *D* is the excess fraction of derived alleles shared between one of two conspecific individuals with a candidate admixing individual (Figure 3). Note that both incomplete lineage sorting (ILS) and admixture can lead to sharing of derived alleles, in this case between polar bears and brown bears. ILS, being a stochastic process, will result in equivalent numbers of shared, derived alleles between any two brown bears and a polar bear. Admixture, on the other hand, will result in more shared, derived alleles in the more admixed bear. Thus, under the null model of no admixture, *D* = 0. A significant non-zero value of *D* indicates more admixture with one of the two individuals.

Comparison of any two polar bears for admixture with brown bears found little evidence for admixture. All *D*-statistics comparing two polar bears to a brown bear were statistically indistinguishable from 0 (Figure 3, top and middle panels).

Conversely, *D*-statistic comparisons between the ABC Islands and mainland brown bears for polar bear admixture were consistently and equivalently non-zero (Figure 3, bottom), regardless of the polar bear used in the comparison (*D* = 0.016, which translates to roughly 0.75% of the genome; *Z*-score = 1.24). Remarkably, when the analysis is restricted to the 12 scaffolds (∼74 Mb of sequence) identified as X-chromosome (Text S1), *D* = 0.22, or ∼6.5% of the X-chromosome (*Z*-score = 4.52) (Figure S2; Tables S3, S4, S5). We find this same enrichment of the X chromosome, compared to the autosome, for admixture with polar bears when analyzing genome sequence data from two additional, recently published ABC Islands brown bears (Figure S3, Table S6) [6]. The ABC Islands bears therefore share not only their mtDNA but also a significant portion of their X-chromosomes with polar bears. A parsimonious explanation for these observations is that the same admixture event that resulted in sharing of the polar bear mtDNA haplotype with ABC Island brown bears also results in sharing of much of the X-chromosome.

**Figure 4:**
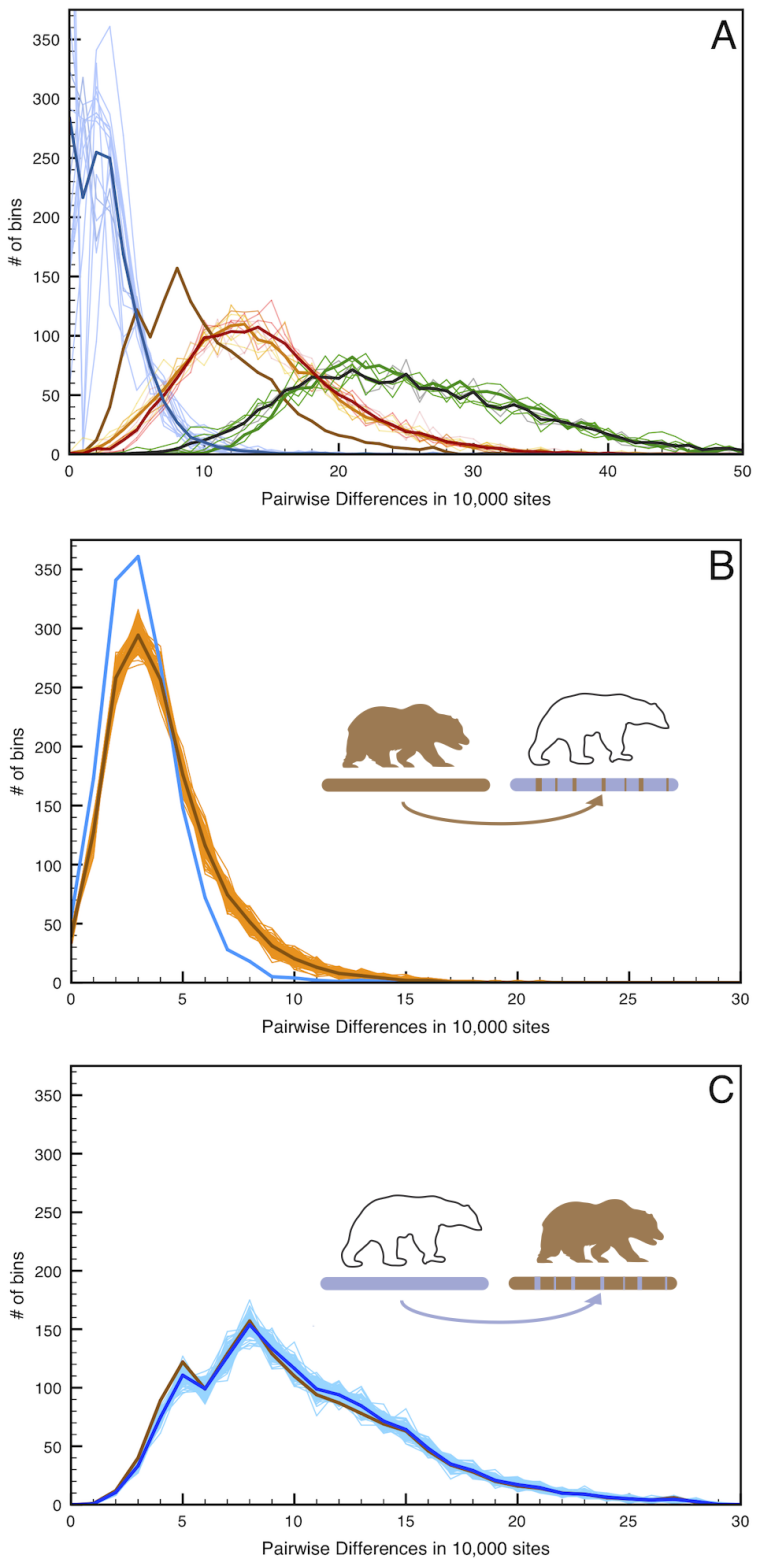
Simulated admixture reveals the direction of gene flow on the X chromosome. (A) Pairwise distance as in Figure 2 but limited to the 12 scaffolds identified as X-chromosome. (B) 100 replicate simulations in which 6.5% of the female West Hudson Bay polar bear X-chromosome is replaced with that of the mainland Alaska brown bear in randomly inserted 20 kb fragments, simulating admixture from the brown bear genome into polar bear ∼50kya. Pairwise differences are calculated between the simulated genome (light brown lines; mean highlighted in dark brown) and the plot comparing the two female polar bears (blue line), to maximize the number of informative sites in the test. The addition of brown bear DNA to the polar bear genome markedly increases the number of high-diversity bins (>10 differences/10 kb), indicating that any introgression of brown bear DNA into polar bears should be easily detectable. (C). As in (B), but with 6.5% of the mainland Alaska brown bear X-chromosome is replaced with that of the female West Hudson Bay polar bear. In this instance, we find no difference between the simulated (blue lines) and real (brown line) data.

To test the direction of X-chromosome gene flow between polar bears and the ABC Islands bear we simulated the effect of having 6.5% ancestry (roughly the amount estimated above) in either polar bear or mainland brown bear X chromosome from the reciprocal species (Figure 4, Figure S5). The simulation was carried out by randomly selecting 6.5% of the X-chromosome of the candidate recipient species to be replaced by sequence from the candidate donor species (Figure S4, Text S1). We then measured the distribution of pairwise divergences that would result following this simulated admixture.

Given the low genetic diversity within polar bears, this amount of brown bear ancestry would be clearly identifiable as an excess of deeply diverging regions between polar bears, even in unphased data from which a random allele is chosen at each site. Conversely, simulating 6.5% polar bear ancestry in the mainland brown bear X-chromosome is more consistent with the observed level of genomic regional divergence between brown bear X-chromosomes. Thus, we deduce that the direction of gene flow was from polar bear into the ABC Islands brown bear X-chromosome.

Recently published genome sequence data from a ∼115ky polar bear [6] allow us to further probe when and in which direction admixture might have happened. Using this ancient polar bear in the *D*-statistic test gives nearly identical results to the extant polar bears (autosome *D* = 0.015; X-chromosome *D* = 0.212). That is, ABC island brown bears are equally enriched for polar bear matching derived alleles, even when this ∼115ky polar bear is used in the comparison. Therefore, *if* the admixture was from the ABC Island brown bears (or a closely related population) into polar bears, it must have occurred prior to ∼115ky. Furthermore, no significant subsequent admixture could have occurred, since the modern polar bears are nearly homogeneous for the ABC Islands brown bear D-statistic signal. Finally, if gene flow was from brown bears into polar bears, it would had to have been from a population of brown bears that lived more than ∼115kya that today finds itself restricted to a group of islands that only became habitable for brown bears since the end of the last glacial maximum, about ∼16kya. Given the unlikeliness of this scenario and the incompatibility of polar bear X-chromosomes genetic divergence with brown bear ancestry, we conclude that the direction of gene flow was from polar bears into the ABC Island brown bears.

## Discussion

The genome-wide analysis presented here indicates that (1) polar bears are a remarkably homogeneous species and show no evidence of brown bear ancestry, (2) the ABC Islands brown bears show clear evidence of polar bear ancestry, and (3) this polar bear ancestry of ABC Islands brown bears is conspicuously enriched in the X-chromosome. ABC Islands brown bears show a simple positive correlation between how maternally biased a genetic locus is (mtDNA>X chromosome>autosomes) and how much polar bear ancestry is present (100%, 6.5%, 1%). Given this observation, and our knowledge about the natural history of these islands through the Pleistocene and Holocene, we present the following model.

During the peak of the last ice age, brown bears were likely absent from the region that now comprises the ABC Islands. Although fossil remains dating to this period are abundant on the more southerly islands of the Alexander Archipelago, brown bears are not among the species present during the period spanning 26-12kya, when glacial conditions were at their peak [17], [18], [19], [20]. Geological and climatological data suggest that if any habitat suitable for brown bears persisted on the ABC Islands during the LGM it would have been limited to the western part of Baranof Island, the most distant of the ABC Islands from the Alaskan mainland [17]. By itself, however, this potential refugium would have been too small to support viable populations of brown bears [21].

Polar bears, alternately, would likely have colonized the sea ice adjacent to the ABC Islands as the ice advanced southward. Notably, marine mammals dominate the fossil remains dating to this interval [19], including ringed seals, an ideal food source for polar bears [2]. As the climate warmed and ice retreated, polar bears may have been stranded on or near the ABC Islands. As the habitat became increasingly hospitable to brown bears [17], the early colonizers from the mainland would have been predominantly the more peripatetic sub-adult males [14]. Admixture involving an influx of mostly or exclusively male brown bears with the stranded polar bears would have resulted in a gradual erosion of the polar bear genome within the isolated population. The sex bias of admixing brown bears would have made genomic erosion more rapid in the autosomes, confining the vestiges of polar bear ancestry in extant ABC Islands bears primarily to matrilineal-biased genetic loci (Figure S12).

Our simplified model - little or no brown bear ancestry in polar bears and matrilineal-biased polar bear ancestry in the ABC Islands brown bears - is consistent with several important comparative genomic observations. First, mtDNA and nuclear genome diversity within both extant and a ∼115kya polar bear is extremely low. This low level of polar bear diversity is consistent with no admixture from brown bears. Brown bears, in contrast, show much higher levels of diversity including many deep genetic lineages that have not completely sorted since their population divergence from polar bears. The ABC Islands brown bears show genome-wide evidence of admixture with polar bears concentrated on the X chromosome. Importantly, the level of admixture inferred from *D*-statistic analyses is only compatible with polar bear admixture into the ABC Islands brown bear X chromosomes and not the other way around. Conveniently, this model explains the presence of the polar bear mtDNA haplotype in all ABC Islands brown bears: the mtDNA haplotype of the male brown bear immigrants is lost, regardless of how many male brown bear immigrants arrived.

The model for historic admixture proposed here is distinct from the traditional framework for admixture, including the scenario involving early humans and Neandertals for which the *D-*statistic analysis was originally developed [16], [22]. Usually, the goal is to find the signal of a potentially small amount of admixture from a single or few admixture episodes that took place many generations ago (Figure S8). While such a model is consistent with the ABC Islands brown bear autosomal *D-*statistic results, it is insufficient to explain the large difference in the X-chromosome or the fixation of the polar bear mtDNA haplotype in the ABC Islands brown bears (Text S1). In fact, reasonable parameter values for a model that assumes a single episode of admixture from polar bears into brown bears do not result in a ratio of *D* for the X and autosomes that exceeds 2.7; our observed ratio is ∼14. Alternately, a long process of sex-biased immigration of brown bears into what was initially a polar bear population can result in much higher ratios of polar bear ancestry for the X and autosomes (Table S8; Figures S9, S10, S11), consistent with the empirical observations presented here.

Spatially explicit modeling has been used to probe the dynamics of gene flow from introgression during species expansions [23]. These simulations have yielded insight into the often non-intuitive patterns seen in various loci such as the apparent asymmetry in gene flow from the native species into the invading species. An extension of this approach to incorporate a migration barrier to female, but not male, gene flow and a dwindling native population of polar bears, may more fully reveal the demographic details of the brown bear invasion. Of particular note, there is evidence that brown bear migration between the mainland and ABC Islands may be ongoing. Analysis of variation at 17 rapidly evolving microsatellite loci indicated that brown bears from Admiralty Island, the closest of the ABC Islands to the mainland, are more similar to mainland Alaskan brown bears than were bears from Baranof and Chichagof Islands [14]. Assuming no disruption of the salient features of this migration, its final state, which has not yet been realized, would be complete conversion of the population, i.e., the fixation of brown bear alleles in all genomic loci in the ABC Island bears except the strictly maternal mtDNA.

We note that our data cannot resolve the *timing* of the origin of polar bears as a distinct lineage. Such an estimate has been hindered mainly by the paucity of preserved ancient polar bear remains [5], [24], and consequent lack of fossil calibrations. However, our data do provide insight into the *relative timing* of divergence between the three bear lineages sampled here. To generate a hypothetical scenario for the timing of the origin of polar bears, we apply several previously suggested calibration strategies to our data (Table 1; Figure 2B). Regardless of the calibration strategy applied, our data support a long interval between the initial divergence between black bears and the brown bear/polar bear lineage, and the later divergence between brown bears and polar bears. This is similar to that observed by Hailer *et al* [4], and in contrast to the scenario predicted by the model of Miller *et al* [6].

**Table 1:** Estimates of genomic TMRCA.

	Scaled	Method A	Method B	Method C
giant panda/black bear	5.99	*8–16 Mya*	23.3–38.8 Mya	12.0 Mya
black bear/brown bear	*1*	1.34–2.67 Mya	*3.90–6.48 Mya*	2.00 Mya
brown bear/polar bear	0.6	0.80–1.60 Mya	2.43–3.89 Mya	1.20 Mya
brown bears (population)	0.43	0.57–1.15 Mya	1.68–2.79 Mya	0.86 Mya
polar bears (population)	0.1	0.13–0.27 Mya	0.39–0.65 Mya	0.19 Mya

Estimates of average genomic TMRCA for black, brown and polar bear lineages, and average population TMRCA for brown bears and polar bears estimated from our data, using three calibration methods (calibrated notes are listed in italics). Estimates are scaled based on an average pairwise distance between sampled brown bears and polar bears of 1 (Figure 2A). Method A assumes divergence between the giant panda and polar bear lineage 12±4 Mya [4]. Method B assumes an average TMRCA between brown bears and black bears 3.9–6.48 Mya [33]. Method C assumes a mammalian mutation rate of 1×10^−9^ substitutions/site/year, the basis for the very old estimates presented in [6].

From analysis of the data presented here, we infer that polar bears most likely became a distinct lineage sometime during the Pleistocene. This timing is consistent with previous molecular (Table 1) and morphological [5] estimates. Polar bears and brown bears were clearly established as a morphologically distinct species by at least ∼115kya – the age of the oldest known polar bear fossil [10], [24]. Regardless of this timing, our data suggest that polar bears have remained a small, distinct lineage since their origin (Figure S6), with lineage-specific adaptations reinforced by the ecological constraints of their extreme environment (Text S1, Table S7) [6]. Brown bears, in contrast, have had a larger effective population size (Figure S6), with segregating polymorphism that often predates their split with polar bears (Figure 2B).

The process of genomic erosion we propose here may not be unique to the stranded ABC Islands polar bears. Past changes in the distribution of polar ice, for example, may have also stranded polar bears or hybrids on present-day Ireland, explaining the appearance of polar bear mtDNA in the remains of extinct Irish brown bears [3]. Long-term climate change may often strand populations on islands or island-like habitats, such as lakes or mountain plateaus. If these stranded populations then hybridize with closely related immigrants, we predict substantial variability in the apparent level of admixture indicated by *D*-statistics. Furthermore, in the case of sex-biased immigration, the ratio of *D*-statistics for the X and autosomes will be highly dependent on the rate and duration of immigration.

## Materials and Methods

We extracted DNA from nine of the ten bears in a modern DNA laboratory using the DNeasy Blood & Tissue Kit (Qiagen) according to the manufacturer's specifications. The historic Lancaster Sound polar bear (Smithsonian Natural History Museum ID 512133; Table S1) was extracted in a dedicated ancient DNA laboratory at Penn State University that is geographically isolated from modern molecular biology research, using a column-based extraction protocol for ancient DNA [25].

We physically sheared the DNA of the modern bears using a Diagenode Bioruptor UCD-200 instrument. Fifty µl of each of the six modern polar bear extracts were transferred into 1.5 ml tubes and exposed to four rounds of sonication for 7 min, using the energy setting “HIGH” and an “ON/OFF interval” of 30 seconds. To attain a longer insert size, we slightly modified the procedure to include two 7-min rounds and one 5-min round of sonication for the brown bears, black bear, and second round of sequencing for two polar bears (West Hudson Bay X3249106A; and Chukchi Sea UP08.010; Table S1). We then purified and concentrated the extracts using the Agencourt AMPure XP PCR purification kit, according to manufacturer's instructions, and eluted in 20 µl of 1×TE, with 0.05% Tween20. The historic bear sample was already fragmented due to degradation, and was not sonicated.

We prepared indexed Illumina libraries using 15 µl of each extract following the protocol described in [26], with reaction volumes scaled to total volume of 40 µl. To verify final DNA concentration and the distribution of insert sizes, we ran each library on an Agilent 2100 Bioanalyzer. We then sequenced each polar bear on a separate lane of an Illumina HiSeq 2000 instrument using 100 base-pair (bp) paired-end chemistry at the UC Santa Cruz Core Genomics Facility. We sequenced one lane each of the two brown bears, the black bear, and an additional lane for two polar bears (Table S2) using an Illumina HiSeq 2000 instrument with 150-bp paired-end chemistry at the Vincent J. Coates Genomics Sequencing Laboratory at UC Berkeley.

From the Illumina sequence data, we removed the index and adapter sequence and merged paired reads using a script provided by M. Kircher [27]. We then trimmed each read to remove low quality bases by trimming inward from the 3′-end of the read until detecting a base with quality score ≥13 (∼95% confidence). We mapped the resulting data to the draft polar bear genome [13] using *BWA* [28]. We removed duplicated reads created by PCR amplification using *rmdup* program from samtools [29]. We then applied GATK's [30] base quality score recalibration and indel realignment, and performed SNP genotyping across all samples simultaneously using default settings in GATK [31]. Total coverage is shown in Table S2.

## Supporting Information

Figure S1 Pairwise distances between all pairs of bears including the historic bear from Lancaster Sound. Plots show histograms for (A) all autosomal data and (B) X chromosome only. The color scheme matches Figure 2A and Figure 3A from the main text. The Lancaster Sound polar bear data are highlighted in dark blue.
(TIF)

Figure S2 Decay of *D*-statistic downstream of ABBA and BABA sites. ABBA and BABA sites for (mainland brown bear, ABC island bear, polar bear, black bear) imply a specific topology (insets) at that site for the sampled haplotypes. *D*-statistics in the downstream vicinity of this focal SNP are heavily biased in the direction of the original observation, as expected.
(TIF)

Figure S3 Proportion of polar bear ancestry of the ABC Islands brown bears calculated using f. The proportion of polar bear ancestry inferred for the autosomes (dark blue) and X chromosome (light blue) is shown for each ABC Islands brown bear; (A) the Admiralty Island brown bear sequenced in this study, (B) the Admiralty Island brown bear of Miller et al, (C) the Baranof Island brown bear of Miller et al [6]. The bears from Admiralty Island show similar amounts of polar bear ancestry but the amount inferred for the Baranof Island bear is much greater. This may be due to the greater distance from the mainland of Baranof Island limiting brown bear immigration to a greater degree than on the more accessible Admiralty Island. The inverse correlation of X chromosome : autosome ratio and total amount of polar bear ancestry is also consistent with our model of population and genome conversion form polar bears to brown bears via sex biased brown bear introgression (Figure S10).
(TIF)

Figure S4 Simulated introgression. To simulate introgression of the amount predicted from our data, we randomly replace sections of the original sequence, shown in blue, with sequence from the introgressor species, shown in red. When only a single introgressed region covers a site in the reference genome it is considered heterozygous, shown in purple, and is represented by either the introgressed or original sequence with equal probability. If two introgressed regions overlap then it is considered to be homozygously introgressed, as is the case on the right side of this figure and in the red region only introgressor sites are selected to represent the individual for the pairwise difference calculation.
(TIF)

Figure S5 Simulations of brown bear into polar bear admixture of various block lengths. In orange are simulations of 6.5% admixture into polar bears in 10,000-year time intervals from 10Kya to 100Kya. The observed pairwise difference between the two female polar bears in the study is shown in blue. There is no systematic effect from different hypothetical times of admixture and all show the same pattern of increased numbers of highly divergent regions of the X chromosome.
(TIF)

Figure S6 Autosomal population sizes through time as estimated with PSMC. 100 bootstrap replicates are shown for the 5 bears listed. We assume a generation time of 10 years and a mutation rate of 1×10^−9^ substitutions/site/year. Note that individuals of the same species show similar profiles. However, polar bears and brown bear profiles do not converge over the time period shown.
(TIF)

Figure S7 Mitochondrial phylogeny for polar bears, ABC Island brown bears and extinct Irish brown bears. Adapted from Edwards *et al.*
(TIF)

Figure S8 Model of a single episode of admixture from polar bears into the ABC brown bear population. N_3_ denotes the effective population size of the polar bears, N_12_ and N_123_ denote the effective sizes of the ancestral populations. The divergence times between populations are given by *t_P2_*, *t_P3_* and *t_out_*. The time of gene flow and the amount of gene flow are given by *t_GF_* and f.
(TIF)

Figure S9 Model of continuous migration of mainland male brown bears to ABC islands initially populated with polar bears. Migration starts at time *t_GF._* The migration rate per generation is constant and equal to *m.*
(TIF)

Figure S10 Changes in allele frequency through time with immigration. Left scale: Frequency of a polar bear allele for an autosomal locus (black line) and an X-linked locus (blue line) as a function of the time period of ongoing mainland brown bear immigration. Right scale: Ratio of the frequency for X and for the autosome. For this graph the migration rate *m* was set to 0.0083.
(TIF)

Figure S11 Effect of sex biased gene flow on X vs Autosome ratio of D statistics. Distribution of D(ABC, Grizzly, Polar, Panda) calculated from data simulated at 12 independent X-linked scaffolds of length 6 Mb with recombination occurring within each locus at rate of 1×10^−8^ per site. Data were simulated using the same parameters as before, but the strength of the sex-bias varies. The ratio of female migration rate by male migration rate ranges from R = 1 (no sex-bias, blue line) to 0 (extreme sex-bias, red line).
(TIF)

Figure S12 Population conversion/genomic erosion model. The salient features of this model are shown schematically. Starting during the last glacial period (left panel), the region is inhabited by polar bears. As the ice retreats and the oceans rise, islands form, cutting off a polar bear or hybrid population from the mainland. Over time, continuous male-dominated or male-exclusive gene flow converts the island population to be of predominantly brown bear ancestry. The remnants of polar bear ancestry are most prevalent in female-associated loci: the mtDNA and X-chromosome.
(TIF)

Table S1 Sample details.
(DOC)

Table S2 Data collected for this analysis. Whole genome shotgun Illumina sequences were collected from ten bears from the locations listed. Number of reads corresponds to the number of reads that mapped to the draft polar bear genome using BWA. Coverage is estimated by averaging the number of reads that map to each site of the draft polar bear genome, after extensive filtering as described in in section 1.2. For two polar bears, we sequenced an additional Illumina lane to increase coverage. The augmented data set (coverage in parentheses) was used for the analysis described in section 2.5.
(DOC)

Table S3 *D-*statistic and *Z* scores using American black bear as outgroup. Significant deviations from zero are highlighted in bold. Abbreviations are as in Table S2. The Lancaster Sound polar bear is not included in tests as I1 or I2.
(DOC)

Table S4 *D-*statistic and *Z* scores using giant panda as outgroup. Significant deviations from zero are highlighted in bold. Abbreviations are as in Table S2. The Lancaster Sound polar bear is not included in tests as I1 or I2.
(DOC)

Table S5 *D-*statistic and *Z* score for admixture test between brown bears, polar bears and the American black bear. The highest coverage polar bears were selected for this analysis. Abbreviations are as in Table S2.
(DOC)

Table S6 *D*-statistic and *Z* score for admixture test between three ABC Islands brown bear and polar bears, using the American black bear as outgroup. Brown bears *Admiralty* and *Baranof* are the two ABC Islands brown bears recently published by Miller and colleagues, and are labeled according to island of origin. Our ABC Island brown bear is also from Admiralty Island, and is labeled ABC (Adm). Other abbreviations are as in Table S2. Significant deviations from *D = *0 are highlighted in bold. The Lancaster Sound polar bear is not included as either I1 or I2.
(DOC)

Table S7 Candidate genetic regions for polar bear adaptation. The genomic coordinates of each of the 100 lowest Polar Bear Accelerated Regions (PBAR) scoring regions are shown along with the dog genes, if any, that map to these regions.
(DOC)

Table S8 Parameter space.
(DOC)

Text S1 Supplementary materials. Expanded materials, methods and analyses.
(DOC)
