# Supplementary figures and images for: Genomic Evidence for Island Population Conversion Resolves Conflicting Theories of Polar Bear Evolution
Source: PLoS Genet. 2013 Mar 14;9(3):e1003345. doi: 10.1371/journal.pgen.1003345 (PMC3597504; doi:10.1371/journal.pgen.1003345)

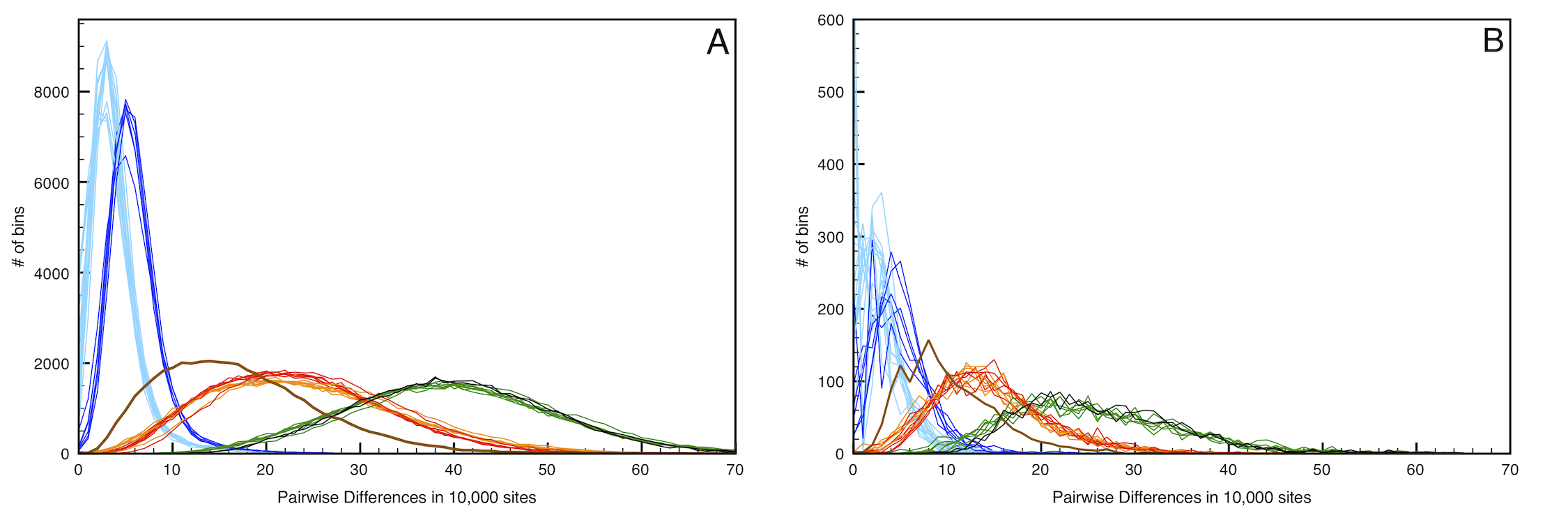

Supplement: Figure S1 — Pairwise distances between all pairs of bears including the historic bear from Lancaster Sound. Plots show histograms for (A) all autosomal data and (B) X chromosome only. The color scheme matches Figure 2A and Figure 3A from the main text. The Lancaster Sound polar bear data are highlighted in dark blue. (TIF) [file pgen.1003345.s001.tif]

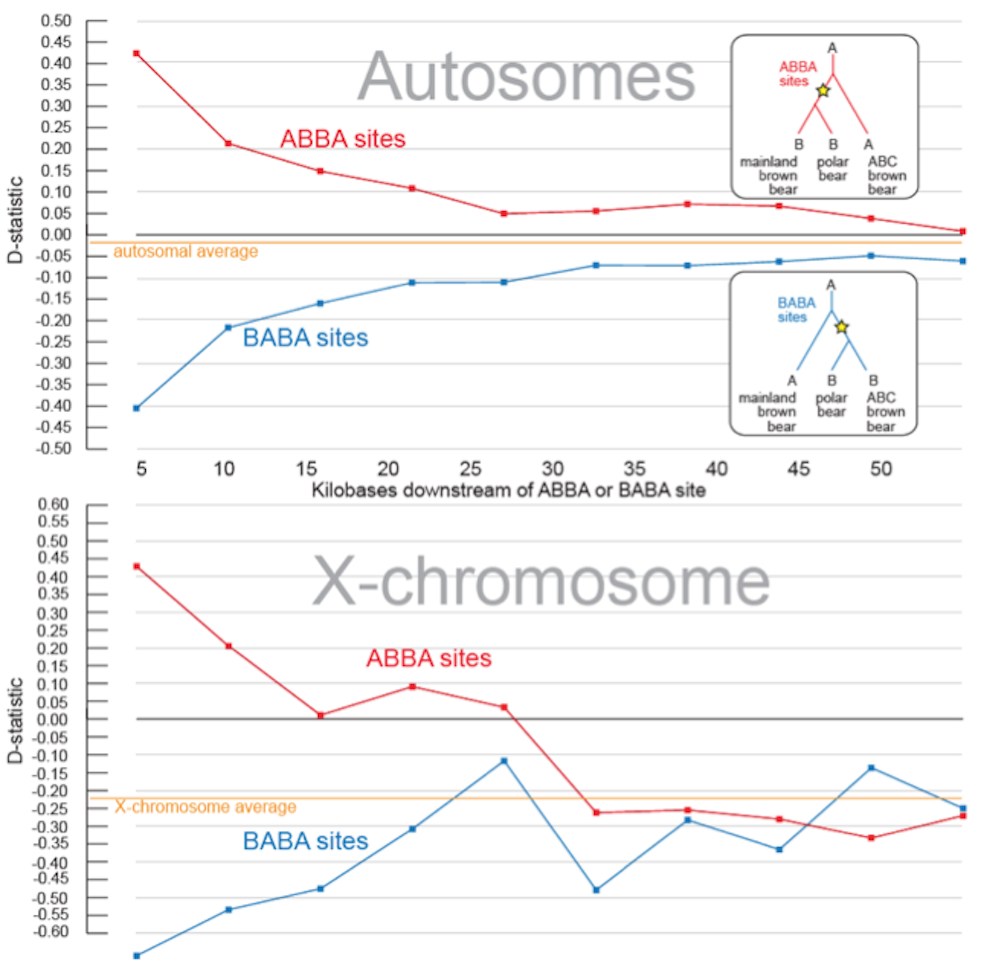

Supplement: Figure S2 — Decay of D-statistic downstream of ABBA and BABA sites. ABBA and BABA sites for (mainland brown bear, ABC island bear, polar bear, black bear) imply a specific topology (insets) at that site for the sampled haplotypes. D-statistics in the downstream vicinity of this focal SNP are heavily biased in the direction of the original observation, as expected. (TIF) [file pgen.1003345.s002.tif]

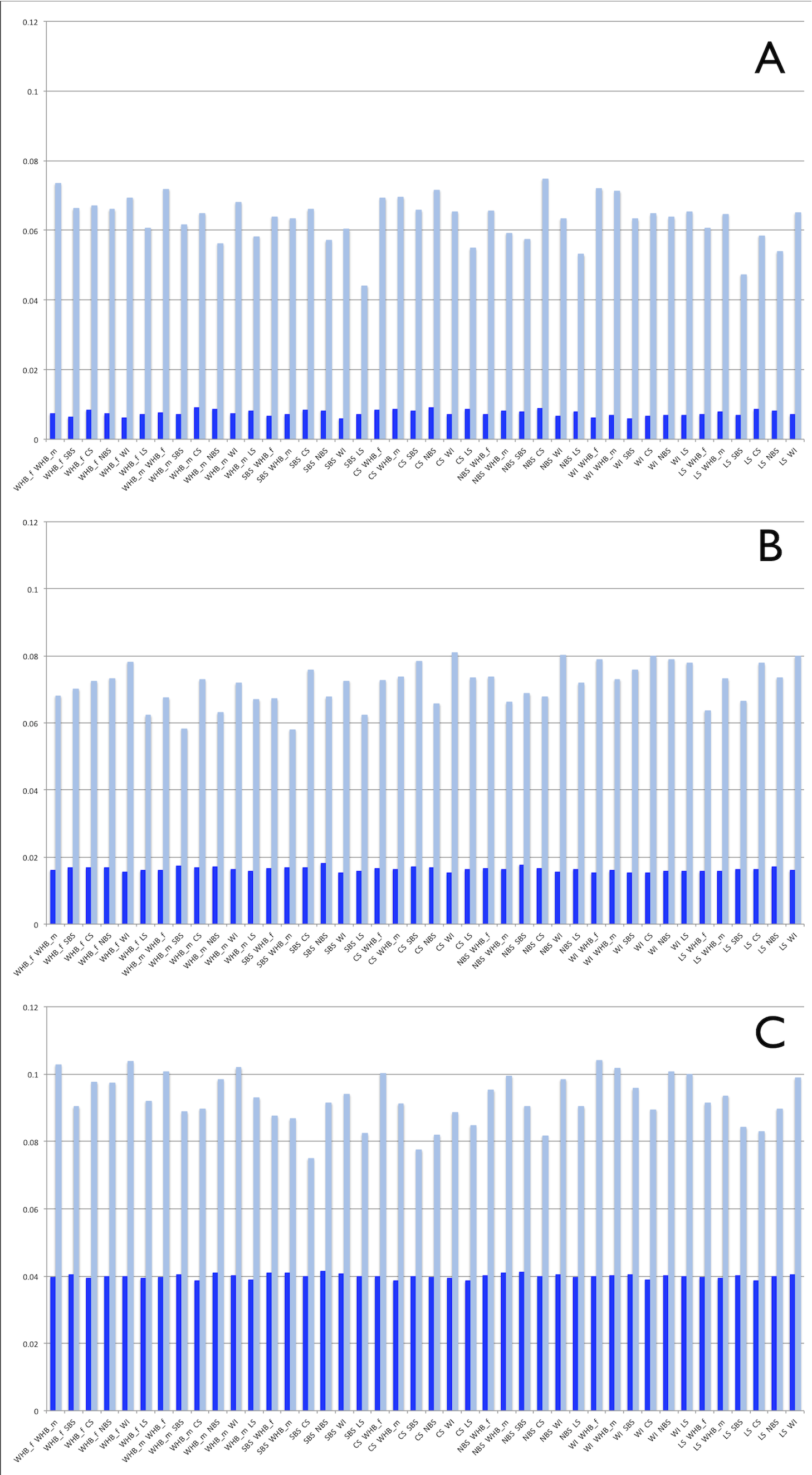

Supplement: Figure S3 — Proportion of polar bear ancestry of the ABC Islands brown bears calculated using f. The proportion of polar bear ancestry inferred for the autosomes (dark blue) and X chromosome (light blue) is shown for each ABC Islands brown bear; (A) the Admiralty Island brown bear sequenced in this study, (B) the Admiralty Island brown bear of Miller et al, (C) the Baranof Island brown bear of Miller et al [6]. The bears from Admiralty Island show similar amounts of polar bear ancestry but the amount inferred for the Baranof Island bear is much greater. This may be due to the greater distance from the mainland of Baranof Island limiting brown bear immigration to a greater degree than on the more accessible Admiralty Island. The inverse correlation of X chromosome : autosome ratio and total amount of polar bear ancestry is also consistent with our model of population and genome conversion form polar bears to brown bears via sex biased brown bear introgression (Figure S10). (TIF) [file pgen.1003345.s003.tif]

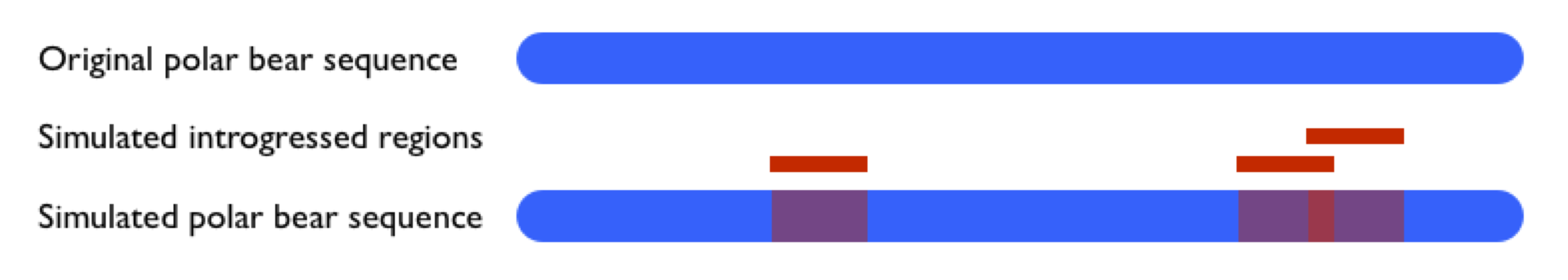

Supplement: Figure S4 — Simulated introgression. To simulate introgression of the amount predicted from our data, we randomly replace sections of the original sequence, shown in blue, with sequence from the introgressor species, shown in red. When only a single introgressed region covers a site in the reference genome it is considered heterozygous, shown in purple, and is represented by either the introgressed or original sequence with equal probability. If two introgressed regions overlap then it is considered to be homozygously introgressed, as is the case on the right side of this figure and in the red region only introgressor sites are selected to represent the individual for the pairwise difference calculation. (TIF) [file pgen.1003345.s004.tif]

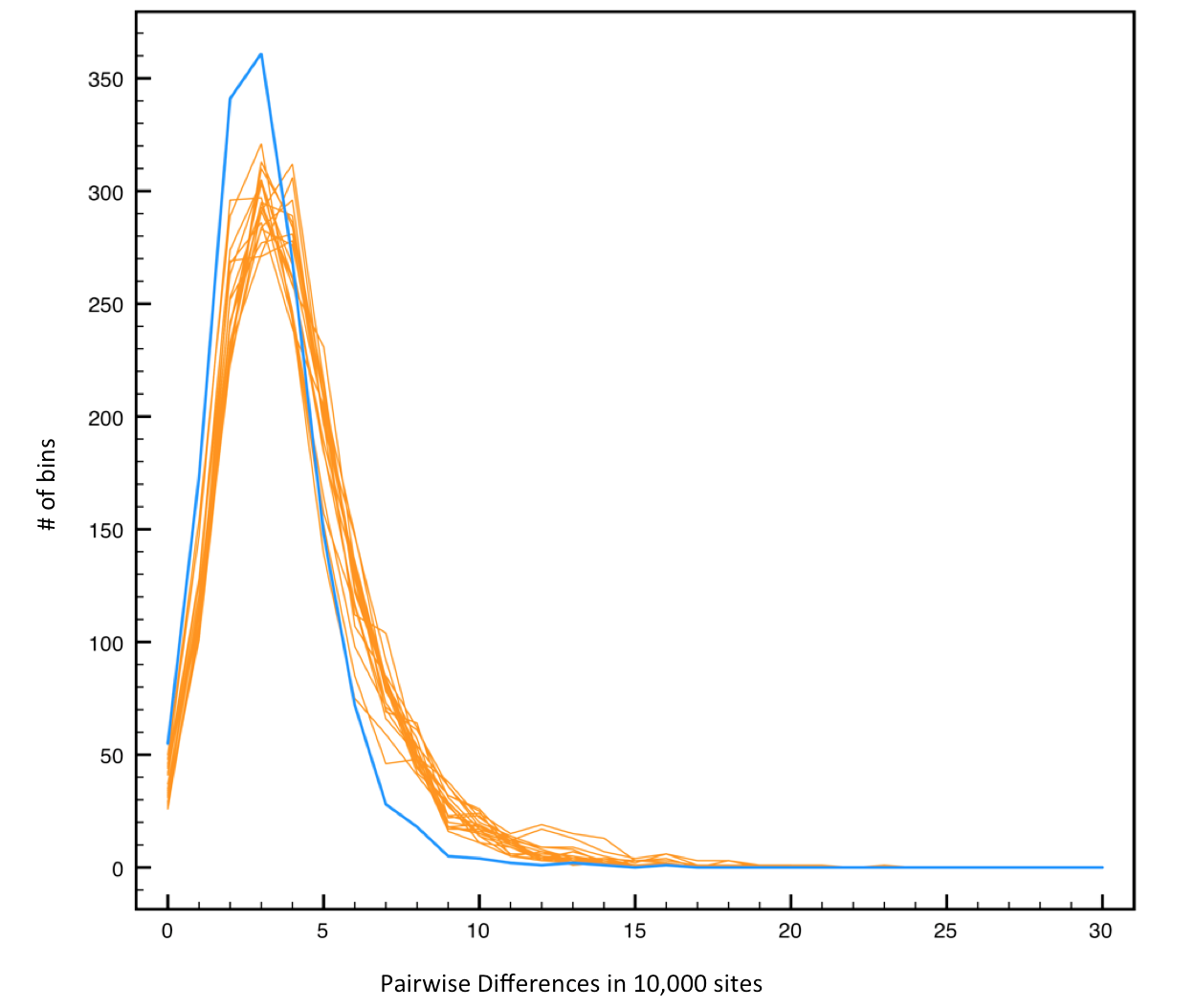

Supplement: Figure S5 — Simulations of brown bear into polar bear admixture of various block lengths. In orange are simulations of 6.5% admixture into polar bears in 10,000-year time intervals from 10Kya to 100Kya. The observed pairwise difference between the two female polar bears in the study is shown in blue. There is no systematic effect from different hypothetical times of admixture and all show the same pattern of increased numbers of highly divergent regions of the X chromosome. (TIF) [file pgen.1003345.s005.tif]

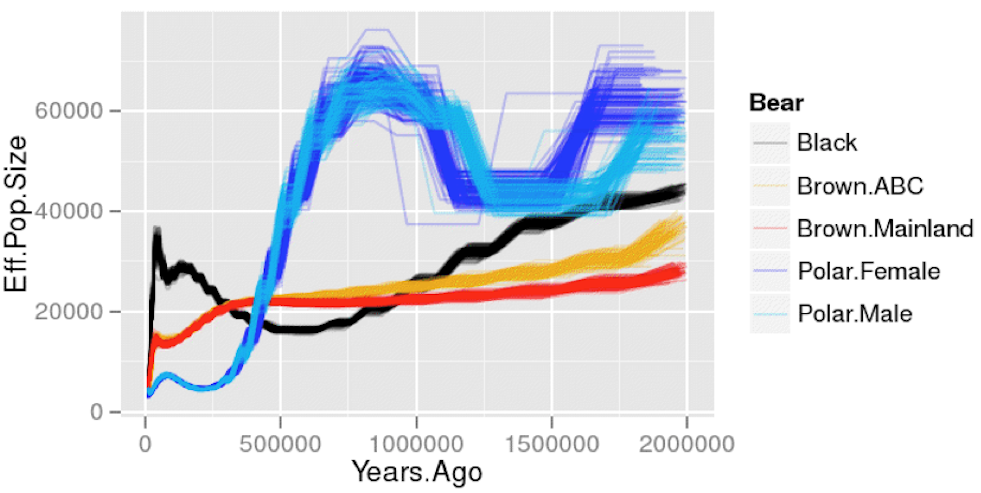

Supplement: Figure S6 — Autosomal population sizes through time as estimated with PSMC. 100 bootstrap replicates are shown for the 5 bears listed. We assume a generation time of 10 years and a mutation rate of 1×10^−9^ substitutions/site/year. Note that individuals of the same species show similar profiles. However, polar bears and brown bear profiles do not converge over the time period shown. (TIF) [file pgen.1003345.s006.tif]

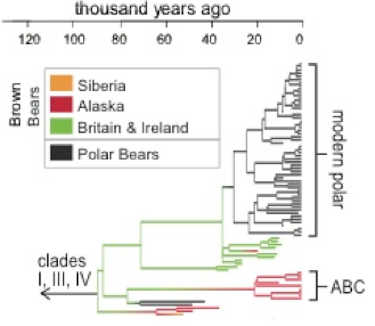

Supplement: Figure S7 — Mitochondrial phylogeny for polar bears, ABC Island brown bears and extinct Irish brown bears. Adapted from Edwards et al. (TIF) [file pgen.1003345.s007.tif]

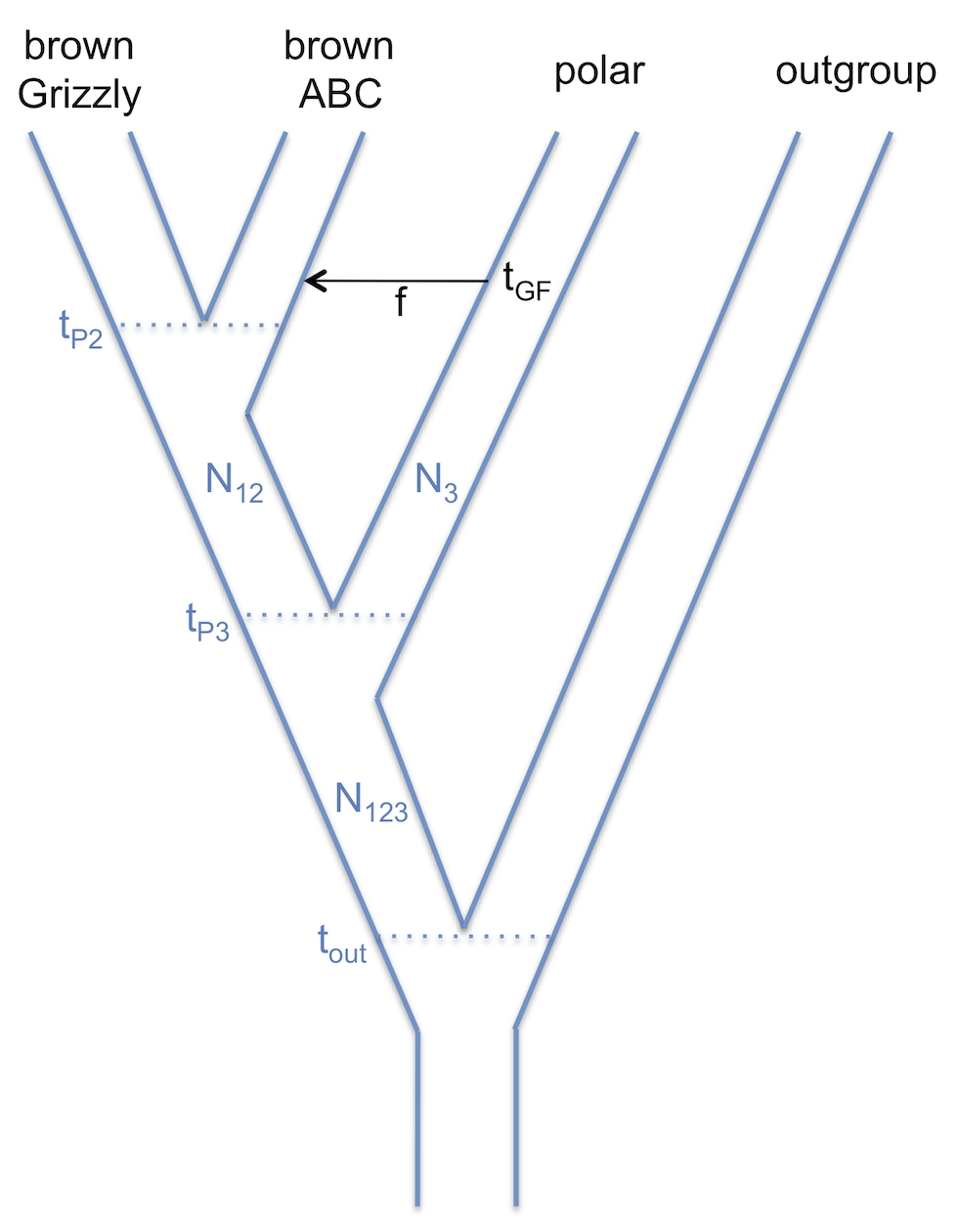

Supplement: Figure S8 — Model of a single episode of admixture from polar bears into the ABC brown bear population. N3 denotes the effective population size of the polar bears, N12 and N123 denote the effective sizes of the ancestral populations. The divergence times between populations are given by tP2, tP3 and tout. The time of gene flow and the amount of gene flow are given by tGF and f. (TIF) [file pgen.1003345.s008.tif]

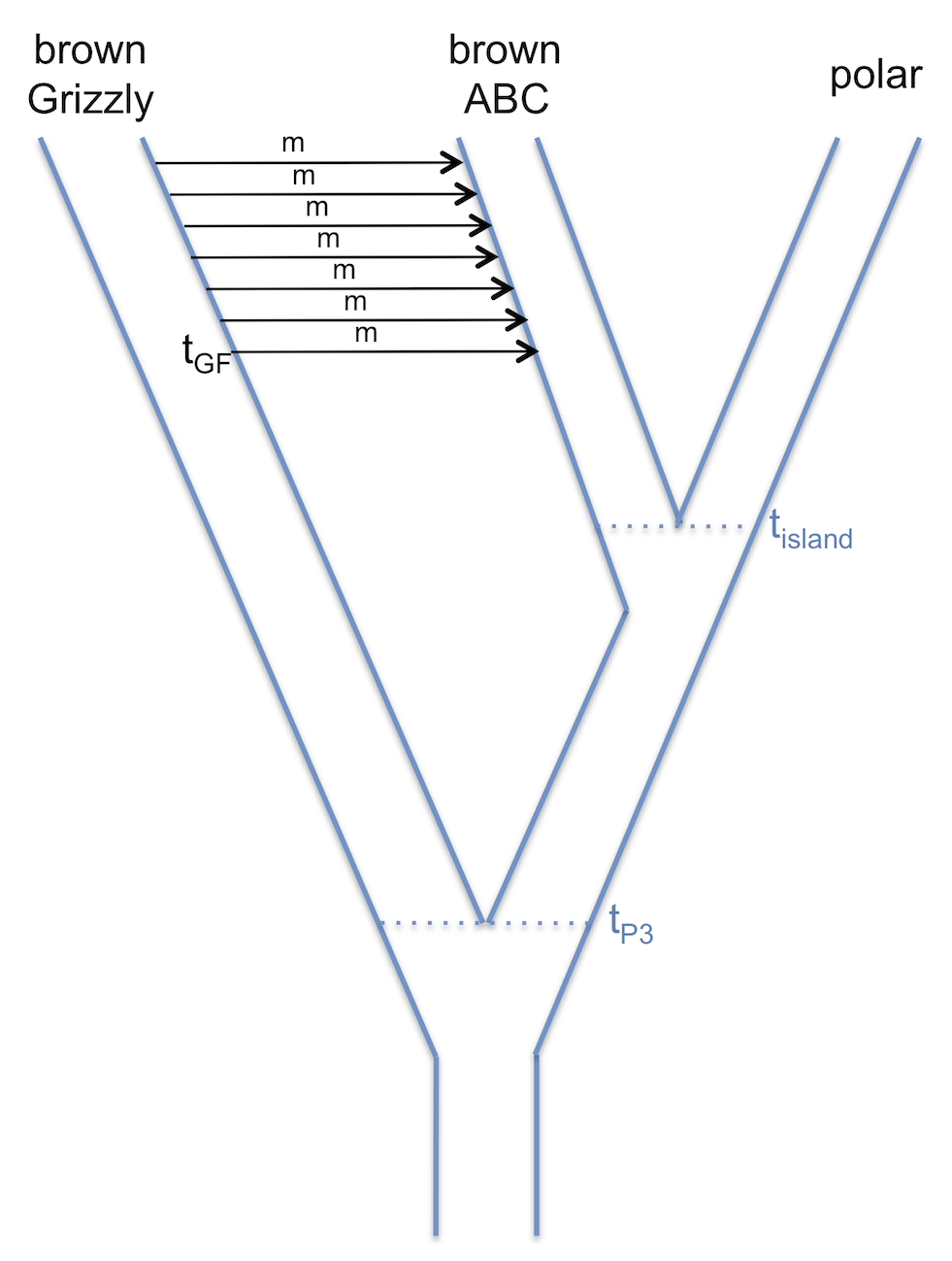

Supplement: Figure S9 — Model of continuous migration of mainland male brown bears to ABC islands initially populated with polar bears. Migration starts at time tGF. The migration rate per generation is constant and equal to m. (TIF) [file pgen.1003345.s009.tif]

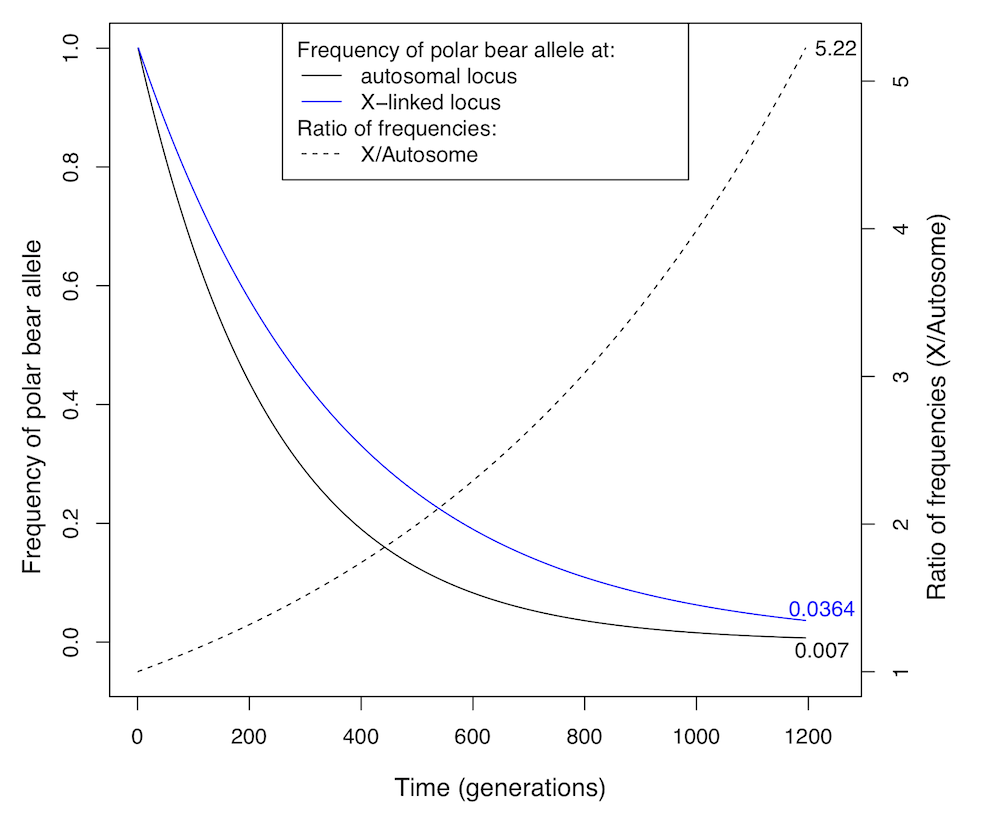

Supplement: Figure S10 — Changes in allele frequency through time with immigration. Left scale: Frequency of a polar bear allele for an autosomal locus (black line) and an X-linked locus (blue line) as a function of the time period of ongoing mainland brown bear immigration. Right scale: Ratio of the frequency for X and for the autosome. For this graph the migration rate m was set to 0.0083. (TIF) [file pgen.1003345.s010.tif]

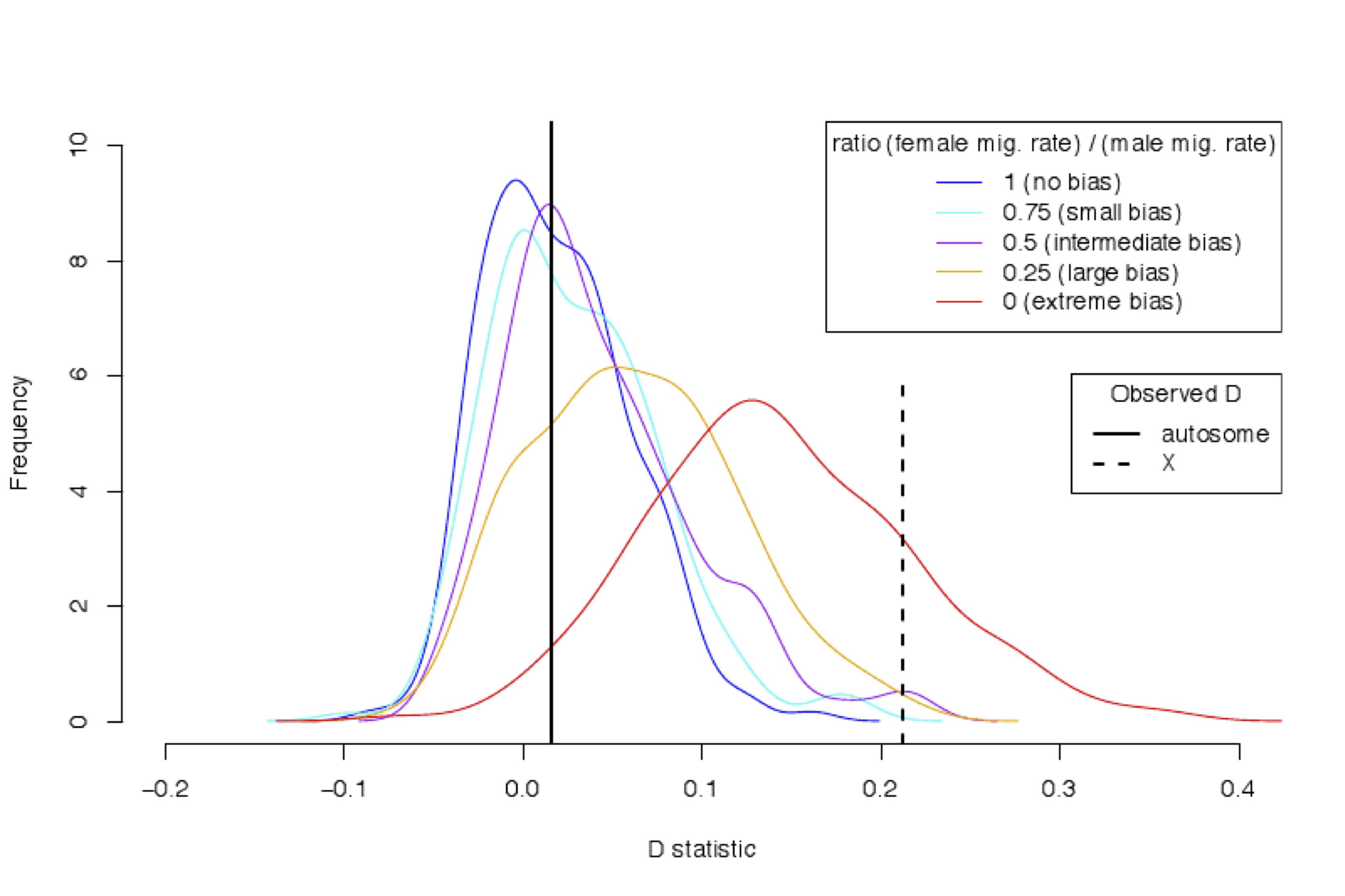

Supplement: Figure S11 — Effect of sex biased gene flow on X vs Autosome ratio of D statistics. Distribution of D(ABC, Grizzly, Polar, Panda) calculated from data simulated at 12 independent X-linked scaffolds of length 6 Mb with recombination occurring within each locus at rate of 1×10^−8^ per site. Data were simulated using the same parameters as before, but the strength of the sex-bias varies. The ratio of female migration rate by male migration rate ranges from R = 1 (no sex-bias, blue line) to 0 (extreme sex-bias, red line). (TIF) [file pgen.1003345.s011.tif]

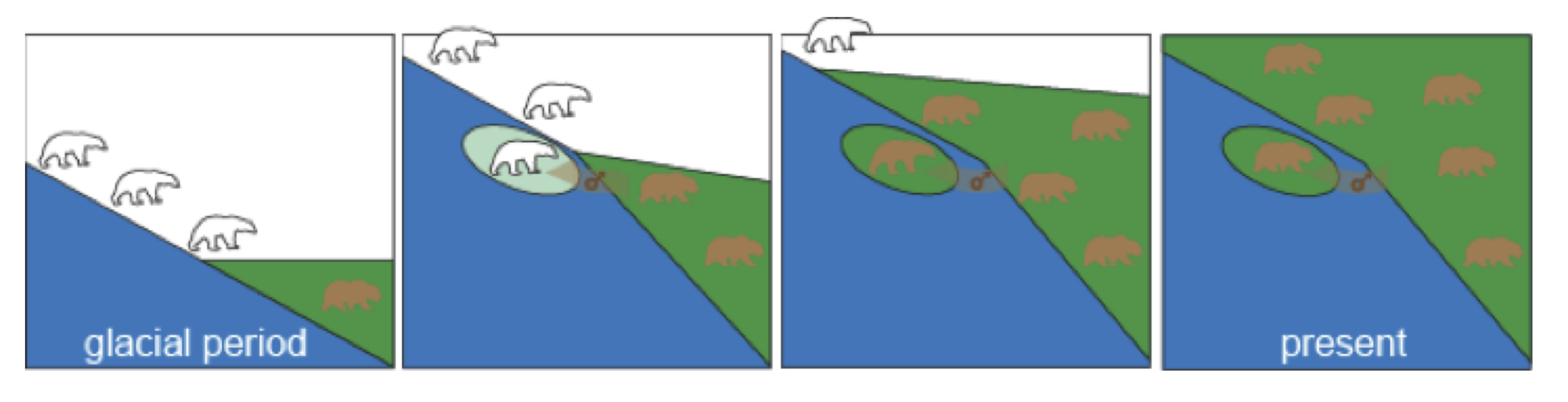

Supplement: Figure S12 — Population conversion/genomic erosion model. The salient features of this model are shown schematically. Starting during the last glacial period (left panel), the region is inhabited by polar bears. As the ice retreats and the oceans rise, islands form, cutting off a polar bear or hybrid population from the mainland. Over time, continuous male-dominated or male-exclusive gene flow converts the island population to be of predominantly brown bear ancestry. The remnants of polar bear ancestry are most prevalent in female-associated loci: the mtDNA and X-chromosome. (TIF) [file pgen.1003345.s012.tif]
