# Supplementary material for: Genomic Evidence for Island Population Conversion Resolves Conflicting Theories of Polar Bear Evolution
Source: PLoS Genet. 2013 Mar 14;9(3):e1003345. doi: 10.1371/journal.pgen.1003345 (PMC3597504; doi:10.1371/journal.pgen.1003345)
Supplement: Table S1 — Sample details. (DOC) [file pgen.1003345.s013.doc]

| **Species** | **Collection** | **Accession number** | **Geographic origin** | **Gender** |
| --- | --- | --- | --- | --- |
| *U. maritimus* | Canadian Wildlife Service; Edmonton, Alberta, Canada | X3249106A | West Hudson Bay | female |
| *U. maritimus* | Canadian Wildlife Service; Edmonton, Alberta, Canada | X3312806A | West Hudson Bay | male |
| *U. maritimus* | Canadian Wildlife Service; Edmonton, Alberta, Canada | X3292306A | North Beaufort Sea | male |
| *U. maritimus* | United States Fish and Wildlife Service; Anchorage, Alaska, USA | 990083KD | North Beaufort Sea | male |
| *U. maritimus* | United States Fish and Wildlife Service; Anchorage, Alaska, USA | 940090KB | Chukchi Sea | male |
| *U. maritimus* | Smithsonian Natural History Museum; Washington DC, USA | 512133 | Lancaster Sound | male |
| *U. maritimus* | Wrangel Island State Nature Reserve | UP08.010 | Wrangel Island | female |
| *U. arctos* | University of Alaska Museum of Natural History; Fairbanks, Alaska, USA | UAM63857 | Admiralty Island, Alaska | female |
| *U. arctos* | University of Alaska Museum of Natural History; Fairbanks, Alaska, USA | UAM33812 | Wood River, Denali National Park, Alaska | female |
| *U. americanus* | Pennsylvania Game Commission, Jersey Shore, Pennsylvania, USA | JC012 | Central Pennsylvania | female |
