# Supplementary material for: Genomic Evidence for Island Population Conversion Resolves Conflicting Theories of Polar Bear Evolution
Source: PLoS Genet. 2013 Mar 14;9(3):e1003345. doi: 10.1371/journal.pgen.1003345 (PMC3597504; doi:10.1371/journal.pgen.1003345)
Supplement: Table S2 — Data collected for this analysis. Whole genome shotgun Illumina sequences were collected from ten bears from the locations listed. Number of reads corresponds to the number of reads that mapped to the draft polar bear genome using BWA. Coverage is estimated by averaging the number of reads that map to each site of the draft polar bear genome, after extensive filtering as described in in section 1.2. For two polar bears, we sequenced an additional Illumina lane to increase coverage. The augmented data set (coverage in parentheses) was used for the analysis described in section 2.5. (DOC) [file pgen.1003345.s014.doc]

| **Species** | **Sampling Location (Abbreviation)** | **Number of reads** | **Coverage** | **Gender** |
| --- | --- | --- | --- | --- |
| Polar bear | Chukchi Sea (CS) | 1.24E+08 | 4.3X (15.8X) | Male |
| Polar bear | Wrangel Island (WI) | 1.90E+08 | 4.7X | Female |
| Polar bear | West Hudson Bay (WHB_f) | 1.69E+08 | 4.9X (17.9X) | Female |
| Polar bear | West Hudson Bay (WHB_m) | 1.50E+08 | 4.5X | Male |
| Polar bear | North Beaufort Sea (NBS) | 1.86E+08 | 5.7X | Male |
| Polar bear | South Beaufort Sea (SBS) | 1.44E+08 | 4.7X | Male |
| Polar bear | Lancaster Sound (LS) | 1.78E+08 | 4.8X | Male |
| Brown bear | ABC Islands (Admiralty Island) (ABC) | 3.54E+08 | 12.1X | Female |
| Brown bear | Denali NP, Alaska (Grizzly) | 3.61E+08 | 12.1X | Female |
| American black bear | Pennsylvania (Black) | 3.28E+08 | 11.6X | Male |
