# Supplementary material for: Genomic Evidence for Island Population Conversion Resolves Conflicting Theories of Polar Bear Evolution
Source: PLoS Genet. 2013 Mar 14;9(3):e1003345. doi: 10.1371/journal.pgen.1003345 (PMC3597504; doi:10.1371/journal.pgen.1003345)
Supplement: Table S3 — D-statistic and Z scores using American black bear as outgroup. Significant deviations from zero are highlighted in bold. Abbreviations are as in Table S2. The Lancaster Sound polar bear is not included in tests as I1 or I2. (DOC) [file pgen.1003345.s015.doc]

| **I1 bear** | **I2 bear** | **M bear** | **O bear** | ***D* (auto.)** | ***Z* (auto.)** | ***D* (X)** | ***Z* (X)** |
| --- | --- | --- | --- | --- | --- | --- | --- |
| ABC | Grizzly | WHB_f | Black | -0.014 | -1.176 | **-0.228** | **-4.149** |
| ABC | Grizzly | WHB_m | Black | -0.016 | -1.272 | **-0.235** | **-7.021** |
| ABC | Grizzly | SBS | Black | -0.014 | -1.119 | **-0.204** | **-3.449** |
| ABC | Grizzly | CS | Black | -0.017 | -1.399 | **-0.223** | **-3.777** |
| ABC | Grizzly | NBS | Black | -0.017 | -1.330 | **-0.207** | **-3.483** |
| ABC | Grizzly | WI | Black | -0.014 | -1.107 | **-0.227** | **-6.135** |
| ABC | Grizzly | LS | Black | -0.016 | -1.279 | **-0.200** | **-3.646** |
| WHB_f | WHB_m | ABC | Black | -0.022 | -1.143 | -0.051 | -0.188 |
| WHB_f | SBS | ABC | Black | -0.014 | -0.676 | 0.200 | 0.874 |
| WHB_f | CS | ABC | Black | -0.005 | -0.228 | -0.175 | -0.569 |
| WHB_f | NBS | ABC | Black | -0.013 | -0.600 | 0.060 | 0.868 |
| WHB_f | WI | ABC | Black | 0.001 | 0.058 | -0.107 | -0.400 |
| WHB_m | SBS | ABC | Black | -0.003 | -0.157 | 0.143 | 0.498 |
| WHB_m | CS | ABC | Black | 0.009 | 0.405 | -0.030 | -0.053 |
| WHB_m | NBS | ABC | Black | 0.011 | 0.596 | -0.027 | -0.117 |
| WHB_m | WI | ABC | Black | 0.015 | 0.726 | 0.018 | 0.061 |
| SBS | CS | ABC | Black | 0.013 | 0.560 | -0.290 | -1.086 |
| SBS | NBS | ABC | Black | 0.009 | 0.408 | -0.176 | -0.667 |
| SBS | WI | ABC | Black | 0.011 | 0.538 | -0.158 | -0.457 |
| CS | NBS | ABC | Black | 0.003 | 0.116 | -0.118 | -0.245 |
| CS | WI | ABC | Black | 0.013 | 0.613 | 0.022 | 0.134 |
| NBS | WI | ABC | Black | 0.004 | 0.173 | -0.099 | -0.339 |
| WHB_f | WHB_m | Grizzly | Black | -0.021 | -0.994 | -0.233 | -0.817 |
| WHB_f | SBS | Grizzly | Black | -0.010 | -0.475 | 0.184 | 1.788 |
| WHB_f | CS | Grizzly | Black | -0.003 | -0.145 | 0.000 | 0.000 |
| WHB_f | NBS | Grizzly | Black | -0.015 | -0.730 | 0.051 | 0.342 |
| WHB_f | WI | Grizzly | Black | -0.002 | -0.103 | 0.117 | 0.630 |
| WHB_m | SBS | Grizzly | Black | -0.015 | -0.700 | 0.388 | 0.955 |
| WHB_m | CS | Grizzly | Black | -0.010 | -0.387 | 0.241 | 0.601 |
| WHB_m | NBS | Grizzly | Black | 0.000 | 0.000 | 0.219 | 0.698 |
| WHB_m | WI | Grizzly | Black | 0.012 | 0.573 | 0.237 | 1.184 |
| SBS | CS | Grizzly | Black | 0.013 | 0.522 | -0.556 | -1.956 |
| SBS | NBS | Grizzly | Black | 0.003 | 0.135 | -0.182 | -0.643 |
| SBS | WI | Grizzly | Black | 0.017 | 0.789 | 0.051 | 0.100 |
| CS | NBS | Grizzly | Black | 0.002 | 0.098 | 0.143 | 0.255 |
| CS | WI | Grizzly | Black | 0.007 | 0.307 | -0.020 | -0.124 |
| NBS | WI | Grizzly | Black | 0.013 | 0.641 | -0.067 | -0.275 |
| **Mean Values** | | | | | | | |
| ABC | Grizzly | Any Polar | Black | -0.015 | -1.240 | **-0.218** | **-4.523** |
