# Supplementary material for: Genomic Evidence for Island Population Conversion Resolves Conflicting Theories of Polar Bear Evolution
Source: PLoS Genet. 2013 Mar 14;9(3):e1003345. doi: 10.1371/journal.pgen.1003345 (PMC3597504; doi:10.1371/journal.pgen.1003345)
Supplement: Table S5 — D-statistic and Z score for admixture test between brown bears, polar bears and the American black bear. The highest coverage polar bears were selected for this analysis. Abbreviations are as in Table S2. (DOC) [file pgen.1003345.s017.doc]

| **I1 bear** | **I2 bear** | **M bear** | **O bear** | ***D* (auto.)** | ***Z* (auto.)** | ***D* (X)** | ***Z* (X)** |
| --- | --- | --- | --- | --- | --- | --- | --- |
| ABC | Grizzly | Black | Panda | -0.002 | -0.354 | 0.014 | 0.263 |
| WHB_f | ABC | Black | Panda | 0.002 | 0.364 | -0.010 | -0.314 |
| WHB_f | WI | Black | Panda | 0.004 | 0.243 | -0.018 | -0.086 |
