# Supplementary material for: Genomic Evidence for Island Population Conversion Resolves Conflicting Theories of Polar Bear Evolution
Source: PLoS Genet. 2013 Mar 14;9(3):e1003345. doi: 10.1371/journal.pgen.1003345 (PMC3597504; doi:10.1371/journal.pgen.1003345)
Supplement: Table S6 — D-statistic and Z score for admixture test between three ABC Islands brown bear and polar bears, using the American black bear as outgroup. Brown bears Admiralty and Baranof are the two ABC Islands brown bears recently published by Miller and colleagues, and are labeled according to island of origin. Our ABC Island brown bear is also from Admiralty Island, and is labeled ABC (Adm). Other abbreviations are as in Table S2. Significant deviations from D = 0 are highlighted in bold. The Lancaster Sound polar bear is not included as either I1 or I2. (DOC) [file pgen.1003345.s018.doc]

| **I1 bear** | **I2 bear** | **M bear** | **O bear** | ***D* (auto.)** | ***Z* (auto.)** | ***D* (X)** | ***Z* (X)** |
| --- | --- | --- | --- | --- | --- | --- | --- |
| Admiralty | Baranof | WHB_f | Black | **0.053** | **3.722** | 0.080 | 0.769 |
| Admiralty | Baranof | WHB_m | Black | **0.053** | **3.689** | 0.087 | 0.746 |
| Admiralty | Baranof | SBS | Black | **0.051** | **3.563** | 0.058 | 0.683 |
| Admiralty | Baranof | CS | Black | **0.049** | **3.296** | 0.024 | 0.197 |
| Admiralty | Baranof | NBS | Black | **0.051** | **3.552** | 0.050 | 0.352 |
| Admiralty | Baranof | WI | Black | **0.054** | **3.739** | 0.063 | 0.556 |
| Admiralty | Baranof | LS | Black | **0.052** | **3.543** | 0.078 | 0.667 |
| Admiralty | Baranof | SVB | Black | **0.050** | **3.505** | 0.035 | 0.323 |
| Admiralty | ABC (Adm) | WHB_f | Black | -0.021 | -1.670 | 0.000 | -0.002 |
| Admiralty | ABC (Adm) | WHB_m | Black | -0.019 | -1.490 | 0.040 | 0.252 |
| Admiralty | ABC (Adm) | SBS | Black | -0.022 | -1.673 | -0.029 | -0.183 |
| Admiralty | ABC (Adm) | CS | Black | -0.019 | -1.440 | -0.060 | -0.380 |
| Admiralty | ABC (Adm) | NBS | Black | -0.022 | -1.631 | -0.031 | -0.187 |
| Admiralty | ABC (Adm) | WI | Black | -0.021 | -1.643 | -0.038 | -0.215 |
| Admiralty | ABC (Adm) | LS | Black | -0.019 | -1.417 | 0.002 | 0.014 |
| Admiralty | ABC (Adm) | SVB | Black | -0.020 | -1.528 | 0.012 | 0.071 |
| Admiralty | Grizzly | WHB_f | Black | **-0.035** | **-2.908** | **-0.250** | **-3.357** |
| Admiralty | Grizzly | WHB_m | Black | **-0.036** | **-2.972** | **-0.245** | **-3.483** |
| Admiralty | Grizzly | SBS | Black | **-0.037** | **-3.013** | **-0.244** | **-3.208** |
| Admiralty | Grizzly | CS | Black | **-0.037** | **-2.932** | **-0.270** | **-2.645** |
| Admiralty | Grizzly | NBS | Black | **-0.038** | **-3.122** | **-0.252** | **-4.326** |
| Admiralty | Grizzly | WI | Black | **-0.035** | **-2.947** | **-0.275** | **-3.567** |
| Admiralty | Grizzly | LS | Black | **-0.035** | **-2.889** | **-0.232** | **-2.736** |
| Admiralty | Grizzly | SVB | Black | **-0.038** | **-3.137** | **-0.232** | **-2.370** |
| Baranof | ABC (Adm) | WHB_f | Black | **-0.070** | **-4.868** | -0.099 | -0.995 |
| Baranof | ABC (Adm) | WHB_m | Black | **-0.070** | **-4.690** | -0.086 | -0.996 |
| Baranof | ABC (Adm) | SBS | Black | **-0.071** | **-4.853** | -0.090 | -1.009 |
| Baranof | ABC (Adm) | CS | Black | **-0.066** | **-4.324** | -0.066 | -0.569 |
| Baranof | ABC (Adm) | NBS | Black | **-0.070** | **-4.691** | -0.086 | -0.809 |
| Baranof | ABC (Adm) | WI | Black | **-0.072** | **-4.869** | -0.110 | -0.984 |
| Baranof | ABC (Adm) | LS | Black | **-0.068** | **-4.612** | -0.107 | -1.285 |
| Baranof | ABC (Adm) | SVB | Black | **-0.067** | **-4.490** | -0.018 | -0.215 |
| Baranof | Grizzly | WHB_f | Black | **-0.082** | **-6.260** | **-0.298** | **-3.170** |
| Baranof | Grizzly | WHB_m | Black | **-0.083** | **-6.291** | **-0.312** | **-3.511** |
| Baranof | Grizzly | SBS | Black | **-0.083** | **-6.376** | **-0.267** | **-2.511** |
| Baranof | Grizzly | CS | Black | **-0.080** | **-6.040** | **-0.274** | **-2.645** |
| Baranof | Grizzly | NBS | Black | **-0.084** | **-6.303** | **-0.280** | **-2.447** |
| Baranof | Grizzly | WI | Black | **-0.083** | **-6.332** | **-0.296** | **-3.162** |
| Baranof | Grizzly | LS | Black | **-0.081** | **-6.156** | **-0.285** | **-3.366** |
| Baranof | Grizzly | SVB | Black | **-0.081** | **-6.269** | **-0.255** | **-2.325** |
| WHB_f | WHB_m | Admiralty | Black | -0.013 | -0.595 | -0.115 | -0.434 |
| WHB_f | SBS | Admiralty | Black | -0.002 | -0.100 | 0.196 | 2.149 |
| WHB_f | CS | Admiralty | Black | -0.006 | -0.268 | -0.088 | -0.284 |
| WHB_f | NBS | Admiralty | Black | -0.001 | -0.048 | -0.017 | -0.100 |
| WHB_f | WI | Admiralty | Black | 0.008 | 0.409 | 0.026 | 0.140 |
| WHB_m | SBS | Admiralty | Black | -0.002 | -0.085 | 0.222 | 0.988 |
| WHB_m | CS | Admiralty | Black | 0.002 | 0.085 | 0.091 | 0.364 |
| WHB_m | NBS | Admiralty | Black | 0.023 | 1.177 | -0.026 | -0.104 |
| WHB_m | WI | Admiralty | Black | 0.024 | 1.166 | 0.098 | 0.303 |
| SBS | CS | Admiralty | Black | 0.000 | -0.015 | **-0.394** | **-2.077** |
| SBS | NBS | Admiralty | Black | 0.016 | 0.776 | -0.254 | -1.245 |
| SBS | WI | Admiralty | Black | 0.013 | 0.638 | -0.083 | -0.292 |
| CS | NBS | Admiralty | Black | 0.015 | 0.715 | -0.053 | -0.101 |
| CS | WI | Admiralty | Black | 0.003 | 0.149 | 0.051 | 0.242 |
| NBS | WI | Admiralty | Black | 0.002 | 0.112 | 0.024 | 0.129 |
| WHB_f | WHB_m | Baranof | Black | -0.014 | -0.686 | -0.059 | -0.149 |
| WHB_f | SBS | Baranof | Black | -0.011 | -0.544 | 0.235 | 1.857 |
| WHB_f | CS | Baranof | Black | -0.016 | -0.643 | -0.161 | -0.491 |
| WHB_f | NBS | Baranof | Black | -0.019 | -0.932 | 0.167 | 1.877 |
| WHB_f | WI | Baranof | Black | -0.002 | -0.119 | 0.023 | 0.089 |
| WHB_m | SBS | Baranof | Black | -0.020 | -0.966 | 0.279 | 0.926 |
| WHB_m | CS | Baranof | Black | -0.015 | -0.613 | 0.273 | 1.247 |
| WHB_m | NBS | Baranof | Black | -0.006 | -0.292 | 0.063 | 0.318 |
| WHB_m | WI | Baranof | Black | 0.007 | 0.333 | 0.113 | 0.397 |
| SBS | CS | Baranof | Black | 0.008 | 0.350 | -0.556 | -1.666 |
| SBS | NBS | Baranof | Black | 0.007 | 0.343 | -0.036 | -0.119 |
| SBS | WI | Baranof | Black | 0.013 | 0.654 | -0.091 | -0.414 |
| CS | NBS | Baranof | Black | 0.003 | 0.138 | 0.154 | 0.345 |
| CS | WI | Baranof | Black | 0.015 | 0.702 | -0.050 | -0.205 |
| NBS | WI | Baranof | Black | 0.014 | 0.703 | -0.171 | -0.959 |
| **Mean Values** | | | | | | | |
| Admiralty | Baranof | Any Polar | Black | **0.052** | **3.576** | 0.060 | 0.537 |
| Admiralty | ABC (Adm) | Any Polar | Black | -0.020 | -1.561 | -0.013 | -0.079 |
| Admiralty | Grizzly | Any Polar | Black | **-0.036** | **-2.990** | **-0.250** | **-3.212** |
| Baranof | ABC (Adm) | Any Polar | Black | **-0.069** | **-4.675** | -0.083 | -0.858 |
| Baranof | Grizzly | Any Polar | Black | **-0.082** | **-6.253** | **-0.283** | **-2.892** |
