# Supplementary material for: Genomic Evidence for Island Population Conversion Resolves Conflicting Theories of Polar Bear Evolution
Source: PLoS Genet. 2013 Mar 14;9(3):e1003345. doi: 10.1371/journal.pgen.1003345 (PMC3597504; doi:10.1371/journal.pgen.1003345)
Supplement: Table S7 — Candidate genetic regions for polar bear adaptation. The genomic coordinates of each of the 100 lowest Polar Bear Accelerated Regions (PBAR) scoring regions are shown along with the dog genes, if any, that map to these regions. (DOC) [file pgen.1003345.s019.doc]

| **polar bear scaffold** | **start : end** | **PBAR score** | **Gene Name** |
| --- | --- | --- | --- |
| scaffold120 | 1778000 : 1906000 | 0.0000 |  |
| scaffold33 | 4186000 : 4254000 | 0.0000 |  |
| scaffold65 | 3241000 : 3308000 | 0.0000 |  |
| scaffold23 | 2212000 : 2278000 | 0.0000 |  |
| scaffold15 | 23999000 : 24056000 | 0.0000 |  |
| scaffold15 | 14041000 : 14094000 | 0.0000 |  |
| scaffold62 | 12365000 : 12415000 | 0.0000 | NFKB1 |
| scaffold33 | 879000 : 929000 | 0.0000 |  |
| scaffold101 | 368000 : 418000 | 0.0000 |  |
| scaffold6 | 27302000 : 27378000 | 0.0047 |  |
| scaffold66 | 12141000 : 12200000 | 0.0061 |  |
| scaffold17 | 19851000 : 19905000 | 0.0068 |  |
| scaffold16 | 7384000 : 7437000 | 0.0072 |  |
| scaffold17 | 20451000 : 20504000 | 0.0072 |  |
| scaffold6 | 27302000 : 27498000 | 0.0077 |  |
| scaffold11 | 26036000 : 26086000 | 0.0084 |  |
| scaffold11 | 26040000 : 26090000 | 0.0089 | TTC3 |
| scaffold29 | 1399000 : 1461000 | 0.0090 |  |
| scaffold78 | 5958000 : 6009000 | 0.0094 |  |
| scaffold2 | 15636000 : 15709000 | 0.0095 |  |
| scaffold83 | 5896000 : 5956000 | 0.0097 |  |
| scaffold19 | 6882000 : 6935000 | 0.0098 | SAG S-antigen/ ATG16L1 |
| scaffold13 | 6092000 : 6154000 | 0.0099 | TMEM39B |
| scaffold19 | 6684000 : 6738000 | 0.0099 | USP40 |
| scaffold13 | 6106000 : 6156000 | 0.0100 | KHDRBS1 |
| scaffold33 | 13477000 : 13535000 | 0.0102 | LCA5 |
| scaffold139 | 4232000 : 4293000 | 0.0102 | KIAA0232 |
| scaffold30 | 5340000 : 5396000 | 0.0102 |  |
| scaffold78 | 7593000 : 7643000 | 0.0106 |  |
| scaffold27 | 5780000 : 5831000 | 0.0108 | ANAPC10 |
| scaffold9 | 6803000 : 6853000 | 0.0112 | PIK3R1 |
| scaffold55 | 1305000 : 1367000 | 0.0114 |  |
| scaffold30 | 16056000 : 16111000 | 0.0114 |  |
| scaffold11 | 19774000 : 19829000 | 0.0115 |  |
| scaffold82 | 3569000 : 3636000 | 0.0115 |  |
| scaffold10 | 9791000 : 9844000 | 0.0116 |  |
| scaffold66 | 522000 : 574000 | 0.0119 |  |
| scaffold15 | 23999000 : 24049000 | 0.0120 | KIAA0196 |
| scaffold220 | 268000 : 325000 | 0.0122 | PDSS1 |
| scaffold6 | 35754000 : 35818000 | 0.0123 |  |
| scaffold95 | 3142000 : 3192000 | 0.0128 |  |
| scaffold1 | 20665000 : 20717000 | 0.0131 | EHBP1 |
| scaffold48 | 218000 : 268000 | 0.0131 |  |
| scaffold155 | 1921000 : 1986000 | 0.0132 | SERPIND1 |
| scaffold55 | 2900000 : 2950000 | 0.0132 |  |
| scaffold15 | 11408000 : 11458000 | 0.0133 | EBAG9/ SYBU/ SNPH |
| scaffold60 | 11749000 : 11801000 | 0.0137 |  |
| scaffold140 | 3455000 : 3511000 | 0.0144 |  |
| scaffold21 | 15269000 : 15319000 | 0.0144 | CLVS2 |
| scaffold250 | 188000 : 243000 | 0.0145 |  |
| scaffold75 | 7819000 : 7870000 | 0.0145 |  |
| scaffold14 | 2533000 : 2583000 | 0.0145 |  |
| scaffold5 | 28373000 : 28427000 | 0.0149 | GPHN |
| scaffold84 | 7504000 : 7558000 | 0.0149 | COX10 |
| scaffold1 | 44825000 : 44876000 | 0.0149 |  |
| scaffold43 | 13061000 : 13114000 | 0.0154 | VMP1 |
| scaffold55 | 4615000 : 4665000 | 0.0154 |  |
| scaffold28 | 4113000 : 4164000 | 0.0155 |  |
| scaffold48 | 4000 : 57000 | 0.0156 |  |
| scaffold17 | 8070000 : 8123000 | 0.0159 |  |
| scaffold6 | 35121000 : 35177000 | 0.0160 | ELAC1 |
| scaffold13 | 8710000 : 8767000 | 0.0160 | GMEB1 |
| scaffold48 | 9700000 : 9752000 | 0.0160 |  |
| scaffold181 | 2392000 : 2444000 | 0.0169 |  |
| scaffold45 | 15653000 : 15707000 | 0.0172 | SCMH1 |
| scaffold57 | 3713000 : 3763000 | 0.0174 | PDE10A |
| scaffold55 | 2536000 : 2591000 | 0.0175 | LOC612664 |
| scaffold11 | 3255000 : 3327000 | 0.0175 |  |
| scaffold40 | 8193000 : 8248000 | 0.0180 |  |
| scaffold45 | 14544000 : 14594000 | 0.0180 |  |
| scaffold41 | 8699000 : 8750000 | 0.0182 |  |
| scaffold55 | 4311000 : 4362000 | 0.0182 |  |
| scaffold55 | 2312000 : 2363000 | 0.0182 | PREP |
| scaffold8 | 14356000 : 14406000 | 0.0182 |  |
| scaffold18 | 17382000 : 17439000 | 0.0189 | ASTN1 |
| scaffold75 | 7261000 : 7314000 | 0.0189 |  |
| scaffold155 | 1950000 : 2004000 | 0.0190 | SNAP29 |
| scaffold7 | 512000 : 562000 | 0.0190 |  |
| scaffold13 | 20852000 : 20904000 | 0.0192 |  |
| scaffold82 | 3503000 : 3557000 | 0.0194 |  |
| scaffold81 | 524000 : 574000 | 0.0194 |  |
| scaffold6 | 27439000 : 27491000 | 0.0195 |  |
| scaffold38 | 7100000 : 7150000 | 0.0196 |  |
| scaffold255 | 70000 : 123000 | 0.0197 | KCNT1/ KCNT2 |
| scaffold8 | 25229000 : 25281000 | 0.0203 | C28H10orf22 |
| scaffold5 | 35857000 : 35907000 | 0.0203 |  |
| scaffold133 | 2333000 : 2385000 | 0.0203 |  |
| scaffold12 | 3411000 : 3467000 | 0.0204 |  |
| scaffold67 | 6066000 : 6117000 | 0.0204 |  |
| scaffold14 | 9003000 : 9053000 | 0.0205 |  |
| scaffold1 | 9082000 : 9137000 | 0.0206 | FSHR |
| scaffold57 | 3691000 : 3741000 | 0.0206 |  |
| scaffold5 | 27529000 : 27579000 | 0.0208 |  |
| scaffold45 | 7733000 : 7784000 | 0.0210 |  |
| scaffold71 | 5482000 : 5533000 | 0.0211 |  |
| scaffold8 | 3258000 : 3310000 | 0.0212 |  |
| scaffold131 | 2224000 : 2277000 | 0.0213 | RNGTT |
| scaffold41 | 3223000 : 3273000 | 0.0213 |  |
| scaffold40 | 8204000 : 8254000 | 0.0214 | SLC46A3 |
| scaffold9 | 19506000 : 19556000 | 0.0216 | SH3RF2 |
