# Supplementary material for: Genomic Evidence for Island Population Conversion Resolves Conflicting Theories of Polar Bear Evolution
Source: PLoS Genet. 2013 Mar 14;9(3):e1003345. doi: 10.1371/journal.pgen.1003345 (PMC3597504; doi:10.1371/journal.pgen.1003345)
Supplement: Table S8 — Parameter space. (DOC) [file pgen.1003345.s020.doc]

| Parameter | Description | Range | #sampled |
| --- | --- | --- | --- |
| t_GF_ | Time of gene flow | [5kya, 50kya] | 10 |
| t_P2_ | Divergence time between mainland and ABC brown bears | [60kya, 300kya] | 25 |
| t_P3_ | Divergence time between brown and polar bears | [300kya, 1000kya] | 71 |
| N_3_ | Effective population size of polar bears | [1k, 10k] | 10 |
| N_12_ | Effective population size of the ancestors of Alaskan mainland and ABC brown bears | [10k, 70k] | 7 |
| N_123_ | Effective population size of the ancestors of brown and polar bears | [10k, 70k] | 7 |
