# Supplementary material for: Genomic Evidence for Island Population Conversion Resolves Conflicting Theories of Polar Bear Evolution
Source: PLoS Genet. 2013 Mar 14;9(3):e1003345. doi: 10.1371/journal.pgen.1003345 (PMC3597504; doi:10.1371/journal.pgen.1003345)
Supplement: Text S1 — Supplementary materials. Expanded materials, methods and analyses. (DOC) [file pgen.1003345.s021.doc]

Supplementary Materials:

**1. Data collection**

**1.1 Samples Collected**

To assess the global genomic diversity of extant polar bears, we collected tissue specimens from seven polar bears (*Ursus maritimus*) from across their present-day range and two Alaskan brown bears (*U. arctos*) from the University of Alaska Museum of Natural History, one from the enigmatic ABC Islands population, and the other from the Alaskan mainland (Figure 1, Table S1) for random shotgun sequencing. To learn the ancestral state of polar bear and brown bear alleles, we performed identical random shotgun sequencing on a single American black bear (*U. americanus*), provided by Anthony Ross, North-Central Regional Wildlife Supervisor of the Pennsylvania Game Commission.

**1.2 Data resulting from Illumina Sequencing after quality control and filtering**

Table S2

**1.3 Identifying the X chromosome**

Most analyses described below are of data mapped to the draft polar bear genome assembly . This assembly contains 72214 scaffolds with N50 of 15.9Mb. Of these, we restrict our analysis to the 238 scaffolds >1 Mb in length, for a combined total genome size ~2.2Gb. Importantly, these scaffolds are not anchored to chromosomes. In order to contrast patterns of divergence and admixture from autosomes to X chromosomes, we took the following approach to identify scaffolds from the polar bear genome that are likely X chromosome.

First, since the X chromosome is homologous across mammals, we used the closest, well annotated genome to bears, i.e., the domestic dog to find polar bear genome scaffolds that contain X-linked genes. We mapped 549 X-linked dog genes to the polar bear scaffolds using *genBLASTa* . From this mapping, we counted the number of dog X chromosome genes that mapped to each polar bear scaffold >1Mb in length. Second, we compared the coverage by shotgun sequencing, assuming that scaffolds of X chromosome should have about half the coverage of autosomal scaffolds in males. We identified scaffolds as putatively X if they showed typical genome-wide coverage in the female brown bears but approximately half of the genome-wide coverage in the male black bear. (We chose the male black bear for comparison as it had higher genome coverage than any of the male polar bears). Finally, we combined those tests, and selected scaffolds as deriving from the X chromosome if they met the coverage criteria *and* contained at least 10 dog X chromosome genes. This resulted in 12 scaffolds designated as X-chromosome (20, 100, 105, 113, 115, 122, 134, 141, 167, 170, 179, 184) with a combined length of 73.7 Mb. This is likely nearly half of the polar bear X chromosome, as the dog X chromosome assembly spans 123.9 Mb.

**1.4 Mapping the panda genome to the polar bear genome**

As an alternative for identifying the ancestral allele for polymorphic sites in brown and polar bears, we also used the reference giant panda (*Ailuropoda melanoleuca*) genome, ailMel1 . We split this genome sequence into non-overlapping segments of 256 base-pairs and used *BWA* to map these data to the polar bear genome. We increased the –n option of *BWA* to 24 for increased mapping sensitivity. For each position of the polar bear genome covered by a panda read, 96.5% were covered by exactly one panda read. We selected that panda aligned base if the read’s map-quality was ≥30. For sites covered by two reads (3.4%), we selected one of the two bases randomly; note that in nearly all cases the two bases are identical. Sites covered by three or more reads were excluded from analysis.

**2. Data Analysis**

**2.1 Estimating pairwise distances between individuals**

We calculated the number of pairwise differences between all individuals by selecting, for each individual, a single base call mapped to each position in the 238 scaffolds of >1Mb length from the polar bear draft genome . Base calls were limited to those with phred >61. This effectively limits our analysis to only positions where the forward and reverse reads overlapped and agreed, i.e., where confidence in the base quality is maximal. We subdivided the scaffolds into non-overlapping 50kb windows, and calculated the number of pairwise differences between individuals, normalized by the total number of sites where both individuals met base quality criteria. We generated histograms of divergence in these 50kb windows by calculating the number of differences in 10,000 bases. In nearly all 50kb bins, more than 10,000 sites were observed, so binning artifacts were minimal. However, some comparisons involving male individuals on the X chromosome scaffolds did show binning artifacts because the lower coverage decreased the total number of sites observed in pairs of individuals. In general, X chromosome scaffolds showed far fewer pairwise differences per 10,000 sites than the autosomes, presumably due to the smaller effective population size of the X chromosome.

Figures 2A and 3A of the main text show histograms of pairwise distance estimates calculated for the autosomes and X-chromosome, *excluding* the historic polar bear from Lancaster Sound. This sample was collected from Cornwallis Island in October of 1973, and is currently part of the collection at the National Museum of Natural History (Smithsonian Institution, Washington DC). As expected from historic and ancient samples, the genomic data from this specimen shows an excess (nearly twice as many) C to T and G to A transitions when compared to the polar bear reference genome than the other, modern polar bears. This pattern is likely due to cytosine deamination to uracil, which is the most common form of post-mortem DNA damage . While including the Lancaster Sound polar bear does not significantly influence the results presented here or in the main text, the effect of the excess of damaged sites is clearly visible in these pairwise divergence plots (Figure S1).

**2.2 Calculating the *D-s*tatistic**

We estimated admixture using the *D-*statistic . The implementation here closely follows that described in . Informally, the test is a comparison of sharing of derived alleles between two individuals of the same species, I1 and I2, with a candidate admixing individual, M, of a different species. Derived alleles are defined by using an out group individual, O. In this case, the American black bear or giant panda, where indicated, is used as outgroup. At each position in the genome, a random single allele is chosen from amongst reads that pass various filtering criteria. We filtered: (i) only analyzing map data from scaffolds ≥1Mb, (ii) from genomic sites where overall read coverage was between the 5^th^ and 95^th^ percentile, genome-wide, (iii) base-quality ≥61, (iv) read-map quality ≥10, (v) uniquely mappable 35-mers .

For genomic sites with a suitable available allele from each individual in the comparison, we write the alleles in the following order: I1, I2, M, and O. Designating the allele from O as A (for ancestral) and an alternate allele as B, we restrict our focus to sites of either ABBA or BABA configuration. That is, we consider *only* sites where M is different from O, i.e., the candidate introgressor has a derived allele and this allele is seen in *either* I1 or I2. Counting the number of such sites, we can calculate:

*D* = (ABBA-BABA)/(ABBA+BABA)

ABBA and BABA sites may be generated by one of four phenomena: admixture, incomplete lineage sorting, multiple mutations occurring at the same site, and machine error leading to incorrect identification of alleles. In the absence of significant ancient population structure, one can expect incomplete lineage sorting, multiple mutations at the same site and machine error to be evenly distributed between ABBA and BABA sites. Any imbalance between the number of ABBA and BABA sites is thus attributed to admixture. Note that even when admixture can be detected, the *D-*statistic does not indicate the direction of gene flow.

To establish statistical confidence for the results, a weighted block jackknifing approach was employed with blocks of 5Mb . Because the polar bear genome consists of scaffolds not mapped to chromosomes, we required complete 5Mb blocks within scaffolds. This requirement will over-estimate the variance and thus make our significance test conservative. A *Z*-score is then calculated by taking the absolute value of *D*/standard error, as per . Tables S3 and S4 show the *Z* score for admixture analysis for all configurations of bears using either the black bear (Table S3) or giant panda (Table S4) as outgroup.

We note that when I1 or I2 contains an excess of postmortem damaged sites, these can influence estimates of both *D* and *Z.* In these instances, damage affects a false positive match between the outgroup (O) and the damaged individual, and consequent identification of the non-damaged individual as potentially admixed. This effect increases as the evolutionary distance between I1/I2 and the outgroup increases. We therefore exclude the Lancaster Sound bear from analyses in which it would be either I1 or I2.

Table S5 shows the results of the *D-*statistic test for admixture between the ABC Island and mainland Alaska brown bears and the American black bear, as recently proposed by Miller and colleagues . We find no support for admixture between brown bears and the American black bear using our approach.

Table S6 shows the results of the *D-*statistic test for our ABC Island brown bear (ABC (Adm)) and the two ABC Island brown bears recently sequenced by Miller and colleagues (Admiralty and Baranof) . We generated reference-based alignments for each bear as described above, and selected a random base-call from each site in the reference genome again as described above.

Although the amount of admixture detected on both autosomal and X-chromosome scaffolds is greater for the two additional ABC Islands brown bears than for our ABC Island brown bear, these two bears also show surprisingly large differences between their autosomal and X-chromosome *D-*statistics. Notably, the brown bear from Baranof Island, the most distant island from the Alaskan mainland, appears to have the most polar bear admixture. The other two bears are from Admiralty Island, the closest island to the Alaskan mainland. The observed differences in estimated *D-*statistics may therefore reflect a gradient of admixture on the ABC Islands. Additional sampling of ABC Island bears will be necessary to fully understand the process of admixture and to determine the role geography and stochastic genetic or demographic processes in determining the distribution of residual polar bear DNA in the ABC Islands brown bear population.

Finally, we performed a *D-*statistic test using the genomic data set from a ~115,000 year old polar bear fossil from Poolepynten, Svalbard, Norway . As above, we generated a reference-based alignment of the ancient polar bear and selected a random single base call from each site in the reference genome. Because the ancient polar bear has very low coverage, we decreased the minimum base call confidence cutoff to phred=20.

If the admixture event(s) detectable in the genomes of the ABC Islands brown bears was, in fact, confined to the ABC Islands, we should also see no sign of admixture in the ancient polar bear relative to other polar bears. To test for a different signature of admixture in the ancient polar bear we performed the *D-*statistic test as:

*D(ABC, Grizzly, Ancient Polar Bear, Am. Black Bear)*

We find that the ancient polar bear results in the same pattern of admixture as the modern polar bears (autosome *D*=-0.015; X-chromosome *D*=-0.213). These results support the hypothesis that the observed admixture was confined to the ABC Islands.

To investigate the local decay of the admixture signal from polar bears into brown bears, we performed the following analysis. We chose ABBA and BABA sites in the autosomal and X-chromosome scaffolds and then measured the *D*-statistic downstream of this focal site in 5kb windows extending out to 50kilobases. The ABBA and BABA focal sites were chosen such that windows would not overlap. The results of this analysis, shown in Figure S2, are consistent with two important expectations for local *D-*statistics locally around such sites. First, the presence of an ABBA or BABA site indicates specific tree topology for at least one of the haplotypes at that site. This topology should extend until recombination changes it. Thus, a single ABBA or BABA sites implies more of the same, locally, and a strong skew of the *D-*statistic in the direction of the focal observation. This result is observed for both autosomes and the X-chromosome. Second, recombination away from the ABBA or BABA implied topology should cause the *D*-statistic to regress toward the genome-wide mean at increasing distance from the focal ABBA or BABA site. Again, the results are consistent with our expectation.

**2.3 Determining the Percentage of the Genome Resulting from Admixture:**

We used the *D*-statistic to calculate the proportion of the various genomes that might be derived from admixture using the f estimator . Informally, this estimates the proportion of an admixed genome deriving from admixture by comparing the rate of derived allele sharing as a proportion of that which would result from a completely admixed individual, M2.

*S(I1,I2,M1,O)* is the numerator of the *D-*statistic: ABBA-BABA

f*= (S(I1,I2,M2,O)/S(I1,M1,M2,O))*

We calculated ffor all combinations of two brown bears and two polar bears in our study. When M1 and M2 are members of the same species as is the case here f will underestimate the amount of the genome resulting from admixture (f) by the following amount :

*f* = f (time_of_speciation – time_of_admixture) / time_of_speciation

As the timing of the admixture event predicted here is much more recent than the timing of initial species divergence, we assume that fis a reasonable approximation of *f*.

Although some comparisons of differential admixture between polar bears produced non-zero *D-*statistic values, none were significant via the block jackknife test. We note also that the *number* of ABBA and BABA sites is extremely small when comparing two polar bears. This is a straightforward consequence of the small amount of genetic variation within polar bears as ABBA and BABA sites require that the two polar bears *differ* from one another at that site.

In contrast, the brown bears show a clear and consistent pattern of differential admixture regardless of which polar bear is used as the candidate for admixture. The average fvalues are 0.75% (stdev = 0.094%) admixture on the autosomes and 6.5% (stdev = 0.483%) admixture on the X chromosome, for an average percent ratio of 8.8 (Figure S3).

**2.4 Characterizing the direction of gene flow**

To test the direction of gene flow between polar bears and brown bears, we substituted 6.5% (a close approximation of the amount of admixture estimated for the X-chromosome, see section 2.3) of the female West Hudson Bay polar bear X chromosome with corresponding X chromosome data from mainland Alaska brown bear (Figure 3B, main text) and vice versa (Figure 3C, main text). For each simulation we randomly selected multiple regions of the X chromosome to be replaced by introgression from the opposite species. To capture the approximate size of haplotypes that would be introduced if the admixture occurred 50kya (assuming 1cM/Mb; generation time of 10 years), admixed regions were 20kb in length. To simulate more recent admixture, we used a smaller number of longer regions, and to simulate more ancient admixture, we used a larger number of shorter regions. To maximize resolution, we conducted this test on female bears only.

Because our pairwise difference method (described in Supplementary section 2.1) uses only a single high quality base call to represent each site, we must distinguish in our simulations between heterozygous introgessed regions (the vast majority) and homozygous introgessed regions. Heterozygous introgressed regions are those where randomly selected admixed regions do not overlap; in these regions we select the base call from the original individual and the introgessing individual randomly with equal probability to represent sampling from one introgressed and one nonintrogressed chromosome. For cases where two introgressed regions overlap, we consider the introgression to be homozygous. In such cases we select exclusively from the introgressing individual in creating the simulated introgressed chromosome. Note that because introgressed regions are simulated as occurring independently on each chromosome the sum of the length of all introgressed haplotypes is equal to two times the amount of admixture predicted in section 2.3, in this case 13% of the length of the X chromosome recovered as described in section 1.3.

We then count the number of pairwise differences in non-overlapping 50kb bins between the simulated introgressed chromosome and the actual data from an individual of the same species (as for Figure 2A in the main text) and compare the results to the true data.

Substituting 6.5% of the polar bear X chromosome with brown bear X chromosome results in an excess of highly diverging bins (bins in which the number of pairwise differences are greater than ~8 in 10,000) compared to the real data for all 100 simulations (Figure 3B, main text). In contrast, substituting 6.5% of the mainland brown bear X chromosome with polar bear X chromosome results in no observable difference compared to the real data (Figure 3C, main text).

To test whether the size of the admixture blocks (and associated estimated time of introgression) influences the results, we performed several additional simulations in which block sizes were selected to correspond to admixture occurring in 10,000 year increments spanning the period 10 kya - 200 kya. In all cases, substituting 6.5% of the brown bear X chromosome into polar bears yields an excess of deeply diverging regions beyond what is seen in real polar bear X chromosome sequence data (Figure S5). This suggests that while Figure 2 depicts results with an assumed time of admixture of 50 kya, similar results are expected for any time of admixture in the range 10-200 kya.

Since the higher within-species diversity of brown bears overlaps with the distribution of divergence between polar bears and brown bears (see Figs. 2B, S1), this test is less powerful to detect the presence of admixture of polar bears into brown bears than *vice versa*. Thus, at present we cannot rule out the possibility of polar bear admixture into *all* brown bears. However, these results do argue against polar bears being the *recipient* of admixture, especially the admixture seen in ABC Island brown bear versus mainland brown bear X chromosome comparison. Thus, we conclude that the ABC Island brown bear and the population it represents are the *recipient* and not the *donor* of X-chromosome genetic material.

**2.5 Inferring population size through time using PSMC**

We calculated the effective population size through time of each of the three species in our study using Li and Durban's PSMC (Figure S6) . PSMC utilizes the density of heterozygous sites within a single individual to infer population size through time. Because identification of heterozygous sites is sensitive to the depth of sequencing, we restricted our analysis to just those individuals with at least 10X coverage. These were the two female brown bears, the male American black bear and two polar bears following additional sequencing (see section 1.1, Table S2): the female West Hudson Bay bear and the male Chukchi Sea bear. PSMC was limited to scaffolds mapping to the autosomes.

We note that although the shapes of the curves are similar to those of Miller *et al.* , we did not observe as large an inferred effective population size for brown bears. The lack of converging population size histories between polar bears and brown bears suggests either that these species were separate populations through the time period in which PSMC has resolution (~1,000,000 years using the generation time and mutation rate described above), or that population structure is complicating the inference of population size through time . Similarly, the very large population size of polar bears in the past may reflect either a much larger effective population size or a high degree of past population structure.

**2.6 Identifying genomic regions that may be under adaptive evolution in the polar bear**

We performed a screen for regions of the polar bear genome that are potentially under very strong or recent positive selection. We identified regions of the genome that satisfy all of the following criteria: (i) polar bears have low within-species variation (ii) polar bears are distantly diverged from brown bears, and (iii) brown bears have average amounts of within-species variation. These criteria were designed to identify candidate regions of polar bear adaptation that are now evolving under strong purifying selection in polar bears.

We generated a vcf file sing GATK with all of the individuals in our sample. Then we generated a sliding window of 50kb width across each scaffold in 1kb increments. In each region we calculated the number of variable sites within polar bears, the number of variable sites in brown bears and the number of fixed differences between polar bears and brown bears. Then for regions where there were at least 20 variable sites in brown bears we define a PBAR (polar bear accelerated region) score as (polar bear variable sites / fixed differences). At that point we set an arbitrary score cutoff of 0.0218. We merged all overlapping regions with a score less than the cutoff and assigned the lowest score in the merged region. After merging we selected the 100 lowest-scoring regions for analysis.

Because the polar bear genome is still lacking gene annotation, we used *genBLASTa* to map dog genes from ENSEMBL to the polar bear genome. We then identified genes that intersected with the PBARs. Although many PBARs do not contain known genes or contain genes of unknown function we did identify several genes that are of potential interest in relation to polar bear evolution. These are shown in Table S7. We note that KCNT2 was also identified as a candidate for selection in polar bears by Miller and colleagues.

**2.7 Inferring the timing of the origin of the polar bear lineage**

The evolutionary relationship between brown bears and polar bears has been a contentious issue for over half of a century. The fossil record for polar bears is markedly lacking , due to their preference for arctic shelf ice and continental edges, where remains are unlikely to be preserved over geological time. The two oldest known polar bear fossils date to around the same age: one from Poolepynten, Svalbard, is estimated to have lived around 110-130 kya , and another from Kiøpsvik, Norway, dates to around 115 kya . Both of these are clearly identifiable as polar bears rather than brown bears, placing a strict lower bound on when polar bears first appeared as a distinct lineage.

In 1964, Björn Kurtén concluded that polar bears evolved from a brown bear–like species no earlier than the Mindel glacial stage of the early Middle Pleistocene (ca. 750-675 kya) . He based this estimate on a comparative analysis of allometric growth patterns in the modern polar bear skull with fossil remains from brown bears and cave bears dating to the early Middle Pleistocene. He noted, however, that some morphological characteristics of modern polar bears, specifically tooth patterns, may have continued to evolve until as recently as the last 10-20,000 years, as polar bear have become increasingly specialized in their extreme environment. Kurtén therefore suggested that the divergence between brown and polar bears may also have occurred much more recently. In making this conclusion, he noted that a more recent divergence could explain why the two species continue to produce fertile hybrid offspring both in zoos and in the wild .

The first decade or so of genetic analyses of brown and polar bears focused mainly on maternally-inherited mitochondrial DNA, and provided additional support for the recent-divergence hypothesis. Polar bears and brown bears share a common mitochondrial ancestor within the within the last 110-160 kya , a period that spans the interval from which the two oldest polar bear fossils are known. In addition, the mitochondrial lineage to which all living polar bears belong falls within the diversity of brown bears, with their closest *living* relatives a population of brown bears from the ABC Islands of southeastern Alaska . This lineage (Figure S7), which also includes ancient brown bears from Ireland and Beringia and mitochondrial lineages from the two polar bear fossils is by convention referred to as *mitochondrial clade II* .

Several hypotheses have been proposed to explain the branching order within mitochondrial clade II. Initially, when genetic data were available only from living individuals (ABC Island brown bears and polar bears), it was hypothesized that the ABC Islands brown bears represented a very old brown bear lineage, and that their close relationship to polar bears reflected the divergence between the two species . However, this hypothesis is inconsistent with four lines of evidence.

First, geological data suggest that the ABC islands were mostly overridden by ice during the peak of the last ice age (Last Glacial Maximum, or LGM; ca 26-14 kya) and therefore not habitable by brown bears . This makes it unlikely that a very old lineage would survive, isolated, in this region. Fossil evidence supports the geological data; brown bear fossils are known from Prince of Wales Island (just south of the ABC Islands) both before (two bones dating to 26 and 31 kya) and after (several bones dating to less than 12 kya) the LGM . However, no brown bear bones are found on either Prince of Wales or the ABC Islands during the LGM. Ample fossil remains of ringed seals (*Phoca hispida*), as well as bones from Arctic foxes (*Alopex lagopus*) and Steller sea lion (*Eumetopias jubatus*) recovered from Prince of Wales Island suggest that taphonomic conditions were favorable for preservation during this period.

Second, nuclear microsatellite data show no evidence of restricted gene flow between brown bear populations living on the ABC Islands today and brown bear populations in mainland Alaska . However, this same analysis does identify restricted gene flow between the Kodiak Islands and mainland Alaska, suggesting that if such a restriction did exist it would be observable from the microsatellite data .

Third, the most recent matrilineal ancestor of the present-day brown bear population on the ABC Islands lived 37-10kya , well after morphologically distinct polar bears are known .

Finally, when the data from the two fossil polar bears are included in the mitochondrial tree (Figure S7), both polar bears and brown bears become paraphyletic with respect to each other . These data suggest a different evolutionary scenario is required to explain the mitochondrial phylogeny.

Precisely when polar bears and brown bears initially split remains controversial. Estimates from nuclear DNA range from ~600 kya , in agreement with Kurtén’s allometry-based estimate, to 4-5 Mya . The large amount of uncertainty in these estimates stems mainly from the poor fossil record within the ursid radiation, which has resulted in a lack of an appropriate calibration. The 600kya estimate of Hailer and colleagues was generated by calibrating the rate of molecular evolution using seven fossils, the most proximate of which was the divergence between the giant panda and the polar bear at around 12Mya. The oldest estimate, that of 4-5 Mya from Miller and colleagues , was based on a serial coalescent model that used an evolutionary rate of 10^-9^ substitutions per site per year. We note that our genomic data do not address the lack of a universally adopted fossil calibration. However, we attempt to compile and contrast various approaches as a necessary precursor to integrating our observations.

Miller and colleagues recently presented full-genome shotgun sequencing data to address the issues of the polar bear origin and subsequent hybridization more thoroughly than had been attempted previously . They identified sites in the nuclear genome that were polymorphic among and between polar and brown bears from high-coverage genome sequences of 27 bears, including 3 brown bears, a black bear, and 23 polar bear. They concluded that polar bears and brown bears diverged 4-5 Mya, at approximately the same time as the polar/brown bear lineage diverged from black bears . Interestingly, the model they propose includes a long period of admixture between brown, polar, and black bear lineages, lasting up until around 100-200 kya for the black bear and the brown/polar lineage, and until the present day between brown bears and polar bears . The finding that polar bears and brown bears formed sister lineages (in contrast to the paraphyletic relationship recovered from analysis of mitochondrial DNA) agreed with previous analyses of a small number of nuclear loci . However the very ancient early divergence was much older than previously suggested, either from morphological or other nuclear data .

The American black bear divergence date given by Miller *et al.* is similar to the time to most recent common ancestor (TMRCA) of American black bears, brown bears and polar bears of 3.9-6.48 Mya estimated by Krause and colleagues . This estimate was based only on a mitochondrial phylogeny, but included both extinct and extant bears. However, Krause *et al.*  estimate the TMRCA of polar bears and a non-ABC islands brown bear to be 0.66-1.17 Mya, again suggesting a recent divergence of brown bears and polar bears, and a long evolutionary distance between the divergence of these two lineages from black bears, and the subsequent divergence of these two lineages from each other. While our genomic data cannot confirm a specific time in which these two divergences occurred, they strongly support a long evolutionary distance between these two events, rather than a rapid radiation of all three lineages around the same time.

**2.8 Modeling admixture between ABC Islands brown bears and polar bears**

The *D*-statistics calculated for the four populations: mainland brown bear, ABC Islands brown bear, polar bear and either the panda or black bear as an outgroup, show that the ABC Islands brown bears are more similar than the mainland brown bear to the polar bear, suggesting admixture between ABC Islands brown bears and polar bears. A surprising result was the very large value of *D* for the X chromosome compared to the value for the autosome (hereafter denoted by *D_X_* and *D*_auto_ respectively). The ratio of *D_X_* to *D*_auto_ is 13.98 when the black bear is the outgroup.

Here, we investigate what underlying model of admixture can explain this discrepancy. In particular, we show that a single episode of admixture from polar bears into brown bears is highly improbable, whereas a continuous sex-biased immigration of brown bears to a polar bear population is more likely to account for these observations.

## I. Scenario 1: single episode of admixture from polar bears into the brown bear population.

Single episode of gene flow

Under the simple model of gene flow described in Figure S8, the analytical expectation of the *D*-statistic, *D(mainland, ABC, polar, outgroup),* for autosomal sites can be derived . This expectation is a function of the divergence time between brown bears and polar bears (*t_P3_*), the divergence time between mainland and ABC Islands bears (*t_P2_*), the different population sizes (*N_3_*, *N_12_*, and *N_123_*), and the time and amount of gene flow (*t_GF_* and *f*). Assuming constant population sizes in each side of the tree, the expectation of *D* is given by:

The expectation of *D* for X-linked sites (*E[D_X_]*) can easily be obtained using equation (1) with effective population sizes scaled by ¾.

Single episode and sex-biased gene flow

If we assume a sex-biased process in which female polar bears mate with male brown bears but not the reverse, the expectation of *D* for autosomal sites is unchanged. However, if the admixing individual were female, *D* for X-linked sites will be larger than for autosomal sites because females bring relatively more X chromosomes into the brown bear population, X (*f_X_* > *f*). The parameter *f* is the probability that an autosomal allele is inherited from a migrant polar bear at the time of gene flow:

In the case of completely sex-biased gene flow

Therefore

Similarly, *f_X_* for the X chromosome is given by

The expectation of *D*_X_ in the case of sex-biased migration can thus be obtained from equation (1) by scaling the effective population sizes *N_3_*, *N_12_*, and *N_123_* by ¾ and *f* by 4/3.

Based on the above results, the ratio *E[D_X_]/E[D_auto_]* is larger if there is sex-bias than if there is no sex bias. Consequently, if the sex-biased model fails to predict large ratios, the non sex-biased model will fail as well. For this reason, we focus on the sex-biased model and show that under this model the ratio cannot be as large as 14 for reasonable parameter values.

We set *f* to 0.007 (see section 2.2) and calculated the ratio *D_X_*/*D_auto_* for different set of parameters using the analytical expression. We explored the 6-dimension parameter space defined by *(t_GF_, t_P2_, t_P3_, N_3_, N_12_, N_123_)* as described in Table S8, resulting in 8,697,500 calculated ratios. All values were in the range [1.13, 2.67], and thus much smaller than the observed *D_X_*/*D_auto_* ratio of 14. Using different values of *f* does not change the range substantially.

Conclusion: A simple episode of polar bear gene flow into brown bears cannot explain the discrepancy observed between *D* calculated for autosome and *D* calculated for X, even in case of an extremely sex-biased gene flow.

## II. Scenario 2: continuous migration of mainland male brown bears to the ABC islands.

Deterministic approach

In the second scenario, the ABC islands were initially populated with polar bears exclusively (Figure S9). At some time *t_GF_* in the past, male brown bears started to immigrate from the Alaskan mainland and replace a fraction *m* of the male bears in the ABC Islands at each generation. Assuming random mating on the island, we can obtain the recurrence equations that describe the change in frequency of a “polar bear allele” (PB allele) at an autosomal locus and at an X-linked locus. When studying alleles that are initially population-specific, and under the assumption of independence between sites, the frequencies of the PB allele can be interpreted as the expected percentage of polar bear ancestry on the island for the autosome and the X chromosome respectively. The aim is thus to compare the expected frequencies to the estimates of polar bear ancestry (~0.75% for the autosome, ~6.5% for the X, section 2.2) and to investigate if Scenario 2 can explain a ratio of ancestry percentage as high as that observed in the real data (~8.8).

Starting with a PB allele frequency of 100% in ABC islands bears and 0% in mainland bears, after *t+1* generations of ongoing migration it can be shown that

- the frequency *p_t+1_* of an autosomal PB allele in the island is given by

$$p_{t+1}=(1-\frac{m}{2})\text{ }p_{t}\text{ }(2)$$

- the frequency *g_t+1_* of an X-linked PB allele in the island female bears is given by

$$g_{t+1}=\frac{(1-m)\text{ }g_{t-1}\text{ }+\text{ }g_{t}}{2}$$

- the frequency *h_t+1_* of an X-linked PB allele in the island males is given by

From equation (2) we obtain that the frequency of the PB autosomal allele is given by

$$p_{t+1}=(1-\frac{m}{2})^{t-1}\text{ }(3)$$

Assuming that the migration started 12kya (1200 generations; approximately when the ABC Islands would have again been habitable by brown bears) and is ongoing, and that the present frequency of the PB allele is 0.7%, we obtain from equation (3) that the migration rate *m* is approximately 0.0083. Figure S10 shows the frequencies of the PB autosomal allele and the PB X-linked allele as a function of the time period of undergoing immigration, for *m* set to 0.0083. The X-linked frequency decreases more slowly than the autosomal frequency, and after 1200 generations the ratio between the two frequencies is 5.22. Note that considering different starting times of immigration, and different migration rates (*m* in [0, 0.1]), did not substantially changed the ratio of frequencies (which was always in the range [5.1, 5.3]).

Although the expected ratio is smaller than the observed ratio (~8.8), Scenario 2 (continuous immigration) provides a much better fit to the data than does Scenario 1. Because stochastic variation in the migration and recombination processes, as well as sampling errors, are expected to generate some discrepancy between the expected and observed ratios (especially since the amount of data for X is limited, we further investigate the scenario using simulations.

Stochastic approach

Using the software ms we simulated sequences for a Grizzly Brown bear, an ABC Brown bear and a Polar bear under scenarios of continuous immigration of Brown bear to the ABC islands (Figure S9). We run simulations under non sex-biased, partially sex-biased, and sex-biased gene flow.

We set the demographic parameters to reasonable values *(N_1_=N_123_=30k, N_2_=N_3_=N_23_=4k, t_P3_=6000kya, t_island_=100kya, t_GF_=12kya)* and then calibrated the migration rate so that the *D* statistics calculated from the simulated autosomal data fit the observed autosomal *D(ABC, Grizzly, Polar, Panda)* (-*0.017*). Assuming non sex-biased gene flow, a migration rate of 0.525% (% of island bears coming from mainland at each generation) was found to be appropriate; in case of complete sex-biased gene flow this corresponds to a rate of 0.525x2=1.05% for males and 0% for females. We then simulated 12 independent X-linked loci each of length 6Mb (mimicking the 12 X-linked scaffolds identified as X chromosome), with the demographic parameters scaled for X, and recombination occurring within each locus at rate 10^-8^ per site. The mutation rate per site was set to 5x10^-9^, so that the number of ABBA patterns simulated roughly equals the one in real X-linked data. Dependence between sites is expected to increase the variance of the simulated *D* values.

Figure S11 shows the distribution of the simulated D statistics for the X chromosome under 5 scenarios of continuous immigration with different strengths of sex-bias (200 independent simulations for each). The blue density line corresponds to a scenario with no sex-bias (the ratio of female migration rate by male migration rate is 1), whereas the red line corresponds to an extreme sex-bias (only males migrate). The observed *D* for X (vertical dotted line) is in the range of the *D* values for simulations under extreme sex-biased gene flow (red line), but not in the range of values for simulations under non sex-biased gene flow or partially sex-biased gene flow (blue, cyan, purple, and orange lines).

Conclusion: The scenario of continuous immigration with an extremely large sex-bias from mainland brown bear to ABC islands is a likely scenario of admixture. Figure S10 shows that even an intermediate bias (purple and orange lines) is unlikely to explain the observed D statistic for X.

**2.9 An ecological assessment of our model for the ABC Islands and other island habitats.**

Our model suggests that the genomic composition of today’s ABC Islands bears is the result of a unique combination of ecological, behavioral and climatological circumstances. In contrast to previous models that suggest polar bears originated on the ABC Islands (see section 2.7), our model, proposes that today’s ABC Islands brown bears share a common ancestor very recently, dating at the earliest to within the last glacial period. We note that this model is in agreement with other recently published data , indicating a recent common ancestor for these bears.

We propose that the ABC Islands population began as a population of polar bears, and, through time, was gradually converted into a population of brown bears via a process of genomic erosion. Continuous input of brown bear DNA, mostly or exclusively from male brown bears, eventually replaced most of the original polar bear genome (Figure S12). The ecology and behavior of the two bear species, in particular behavioral differences between males and females, both enabled this process and made it possible to recognize it from the genomic data.

It is notable that the polar bear/brown bear hybrids that are observed in the Canadian Arctic today have exclusively, as far as is known, been born from crosses of polar bear females (or hybrids) and brown bear males . Adult male brown bears are known to emerge from their winter dens prior to the spring snowmelt and move onto sea ice to scavenge seals killed by polar bears. Although the peak breeding season for polar bears lasts from April into early May and for brown bears from the end of May through June, male spermatogenesis in brown bears covers a much wider temporal range, and female polar bears are induced ovulators . Therefore, although their breeding periods do not overlap entirely, the production of hybrids is thus possible. Hybrid cubs will stay with their mothers, likely following the adult females onto the ice until weaning. However, they may be more predisposed to make use of terrestrial habitat, and therefore more vulnerable to “stranding” or at least spending more time on terrestrial habitats as they reach adulthood.

While we cannot know the precise process leading to the stranding or isolation of the colonizing population of polar/hybrid bears on the ABC Islands, we propose the following scenario, based on both ecological and genomic data (Figure S12): As the ice retreated around the ABC Islands toward the end of the last glacial period, polar bears living on the edges of this ice may have come into contact with male brown bears under a scenario similar to that seen today in the Canadian Arctic. Somehow, a population of polar bear or polar/brown hybrids settled on these islands. Over time, sub-adult male brown bears, the main class of dispersing brown bears individuals , dispersed from the mainland to become part of the resident adult population on the ABC Islands (Figure S9). As this happened, the genomes of the bears isolated on the ABC Islands would became more and more brown bear-like, in a process we term *genomic erosion*. This process essentially converts the population that was once polar bears into the population of brown bears observed today. With sufficient time, we expect this process to lead to the complete erosion of the polar bear genome within this population, with the exception of the mitochondrial genome, as no mitochondrial input is received from the dispersing brown bear males.

While we focus here on inferring what may have happened in the ABC Islands, similar processes of hypothetical “stranding” followed by introgression may have occurred in other island or island-like habitats, in particular during periods of climate change as species’ ranges change and temporarily overlap. The resulting genetic imprint will depend on several demographic parameters, including the starting population size, whether the immigrant admixers are restricted to one sex, and how long it has been since the process began. Further genomic assessment, for example of the extinct Irish brown bear population, will help to refine these theories and better understand how natural climate change can determine the distribution and genetic diversity of species.

References

1. Li B, Zhang G, Willerslev E, Wang J, Wang J (2011) Genomic data from the Polar Bear (Ursus maritimus). Available from: http://dx.doi.org/10.5524/100008. GigaScience.

2. Lindblad-Toh K, Wade CM, Mikkelsen TS, Karlsson EK, Jaffe DB, et al. (2005) Genome sequence, comparative analysis and haplotypes structure of the domestic dog. Nature 438: 803-819.

3. She R, Chu JSC, Wang K, Pei J, Chen NS (2009) genBlastA: Enabling BLAST to identify homologous gene sequences. Genome Research 19: 143-149.

4. Li R, Fan W, Tian G, Zhu H, He L, et al. (2010) The sequence and de novo assembly of the giant panda genome. Nature 463: 311-317.

5. Hofreiter M, Jaenicke V, Serre D, von Haeseler A, Paabo S (2001) DNA sequences from multiple amplifications reveal artifacts induced by cytosine deamination in ancient DNA. Nucleic Acids Research 29: 4793-4799.

6. Durand EY, Patterson N, Reich D, Slatkin M (2011) Testing for ancient admixture between closely related populations. Molecular Biology and Evolution 28: 2239-2252.

7. Green RE, Krause J, Briggs AW, Maricic T, Stenzel U, et al. (2010) A Draft Sequence of the Neandertal Genome. Science 328: 710-722.

8. Derrien T, Estelle J, Sola SM, Knowles DG, Raineri E, et al. (2012) Fast Computation and Applications of Genome Mappability. PLoS ONE 7.

9. Miller W, Schuster SC, Welch AJ, Ratan A, Bedoya-Reina OC, et al. (2012) Polar and brown bear genomes reveal ancient admixture and demographic footprints of past climate change. Proceedings of the National Academy of Sciences of the United States of America.

10. Li H, Durbin R (2011) Inference of human population history from individual whole-genome sequences. Nature 475: 493-496.

11. Harington CR (2008) The evolution of Arctic marine mammals. Ecological Applications 18: S23-S40.

12. Ingólfsson Ó, Wiig Ø (2009) Late Pleistocene fossil find in Svalbard: the oldest remains of a polar bear (*Ursus maritimus* Phipps, 1744) ever discovered. Polar Research 28: 455-462.

13. Kurtén B (1964) The evolution of the polar bear, *Ursus maritimus* (Phipps). Acta Zool Fenn 108: 1-26.

14. Davison J, Ho SYW, Bray SC, Korsten M, Tammeleht E, et al. (2011) Late-Quaternary biogeographic scenarios for the brown bear (Ursus arctos), a wild mammal model species. Quaternary Science Reviews 30: 418-430.

15. Kowalska Z (1965) "Cross breeding between a female European brown bear and a male polar bear in Lodz Zoo.". Przeglad Zoologiczny, 9: 313-398.

16. Preuß A, Gansloßer U, Purschke G, Magiera U (2009) Bear-hybrids: behaviour and phenotype. Zool Garten N F 78: 204-220.

17. Stirling I (2011) Polar Bears: The Natural History of a Threatened Species. Brighton, Mass: Fitzhenry and Whiteside.

18. Edwards CJE, Suchard MA, Lemey P, Welch JJ, Barnes I, et al. (2011) Ancient hybridization and a recent Irish origin for the modern polar bear matriline. Current Biology 21: 1251-1258.

19. Lindqvist C, Schuster SC, Sun Y, Talbot SL, Qi J, et al. (2010) Complete mitochondrial genome of a Pleistocene jawbone unveils the origin of polar bear. Proc Natl Acad Sci U S A 107: 5053-5057.

20. Cronin MA, Amstrup SC, Garner GW, Vyse ER (1991) Interspecific and Intraspecific Mitochondrial-DNA Variation in North-American Bears (Ursus). Canadian Journal of Zoology-Revue Canadienne De Zoologie 69: 2985-2992.

21. Shields GF, Kocher TD (1991) Phylogenetic-Relationships of North-American Ursids Based on Analysis of Mitochondrial-DNA. Evolution 45: 218-221.

22. Talbot SL, Shields GF (1996) Phylogeography of brown bears (Ursus arctos) of Alaska and paraphyly within the Ursidae. Molecular phylogenetics and evolution 5: 477-494.

23. Waits LP, Talbot SL, Ward RH, Shields GF (1998) Mitochondrial DNA Phylogeography of the North American Brown Bear and Implications for Conservation. Conservation Biology 12: 408-417.

24. Barnes I, Matheus P, Shapiro B, Jensen D, Cooper A (2002) Dynamics of Pleistocene population extinctions in Beringian brown bears. Science 295: 2267-2270.

25. Leonard JA, Wayne RK, Cooper A (2000) Population genetics of ice age brown bears. Proceedings of the National Academy of Sciences of the United States of America 97: 1651-1654.

26. Heaton TH, Talbot SL, Shields GF (1996) An Ice Age refugium for large mammals in the Alexander Archipelago, southeastern Alaska. Quaternary Research 46: 186-192.

27. Carrara PE, Ager TA, Baichtal JF (2007) Possible refugia in the Alexander Archipelago of southeastern Alaska during the late Wisconsin glaciation. Canadian Journal of Earth Sciences 44: 229-244.

28. Heaton TH, Grady F (2003) The Late Wisconsin vertebrate history of Prince of Wales Island, southeast Alaska. In: Schubert BW, Mead JI, Graham RW, editors. Ice Age Cave Fauna of North America: Indiana University Press. pp. 17-53.

29. Paetkau D, Shields GF, Strobeck C (1998) Gene flow between insular, coastal and interior populations of brown bears in Alaska. Molecular Ecology 7: 1283-1292.

30. Hailer F, Kutschera VE, Hallstrom BM, Klassert D, Fain SR, et al. (2012) Nuclear Genomic Sequences Reveal that Polar Bears Are an Old and Distinct Bear Lineage. Science 336: 344-347.

31. Yu L, Li Q-w, Ryder OA, Zhang Y-p (2004) Phylogeny of the bears (Ursidae) based on nuclear and mitochondrial genes. Molecular phylogenetics and evolution 32: 480-494.

32. Krause DW (2010) Biogeography: Washed up in Madagascar. Nature 463: 613-614.

33. Hudson RR (2002) Generating samples under a Wright-Fisher neutral model of genetic variation. Bioinformatics 18: 337-338.
